# Genetic make-up and regulation of the L-lysine biosynthesis pathway in *Vibrio natriegens*

**DOI:** 10.15698/mic2026.02.867

**Published:** 2026-02-03

**Authors:** Elly Straube, Johannes Radde, Thi Van Anh Tran, Negin Keihani Yazdi, Rubén Crespo Blanco, Ha Thanh Le, Cláudio J.R. Frazão, Thomas Walther

**Affiliations:** 1 Institute of Natural Materials Technology, TU Dresden, 01062 Dresden, Germany; 2 School of Chemistry and Life Science, Hanoi University of Science and Technology, 1 Dai Co Viet, Hanoi, Vietnam

**Keywords:** L-lysine biosynthesis, aspartate-family amino acids, *Vibrio natriegens*, transcriptome analysis

## Abstract

*Vibrio natriegens*, the fastest growing non-pathogenic microorganism known to date, has emerged as a highly promising chassis strain for synthetic biology and biotechnology applications. This study analysed the make-up and regulation of the biosynthetic pathway for L-lysine and related L-aspartate family amino acids (AFAAs) in *V. natriegens* DSM759 to provide a comprehensive basis for future metabolic engineering endeavours aiming at developing this strain into an amino acid overproducer. The compilation of automatically annotated genome sequencing data revealed the presence of gene duplicates encoding putative isozymes for multiple enzymatic reactions within these pathways. The physiological role of these isozymes was analysed via growth phenotyping of corresponding gene deletion mutants as well as enzymatic assays. We verified the presence of a previously unknown mono-functional aspartate kinase isozyme, here termed Vn.LysC2, which was shown to be insensitive to allosteric inhibition by any AFAA. In addition, functional duplicates of L-aspartate semialdehyde dehydrogenase and dihydrodipicolinate synthase enzymes were identified. RNA sequencing experiments were used to elucidate the transcriptional regulation mediated by AFAAs on both their corresponding biosynthetic pathways as well as on the global metabolism. The presence of L-lysine, L-threonine, L-isoleucine and L-methionine resulted in the transcriptional repression of their respective biosynthetic pathways. A global analysis of the transcriptional response revealed that the transcriptional response to L-lysine and L-isoleucine was characterised by a high degree of specificity (four and seven differentially expressed genes, respectively), while L-methionine and L-threonine supplementation affected the expression of a larger number of genes (37 and 60 differentially expressed genes).

## INTRODUCTION

Amino acids are fundamental building blocks of life, with 20 standard amino acids participating in protein synthesis 1. While humans and animals can biosynthesize only eleven of these amino acids, the remaining nine must be obtained through diet or supplements 2. One of these nine essential amino acids is L-lysine, a key member of the L-aspartate family amino acids (AFAAs), which also comprises L-threonine, L-isoleucine, and L-methionine 3–6. With an estimated global market of $1.26 billion in 2023 and an expected annual growth rate of 8.2% from 2024 to 2030, L-lysine is of significant economic value 7. It is widely used in animal feed, food and dietary supplements, pharmaceuticals, as well as cosmetics. It also serves as a precursor in the chemical industry 8. Over the years, L-lysine production has shifted from traditional methods such as chemical synthesis and protein hydrolysis to microbial fermentation employing engineered producer strains including *Escherichia coli* and *Corynebacterium glutamicum* 9, 10. For these producer strains, L-lysine biosynthesis has been investigated in great detail and fermentation processes have been optimized to reach maximum L-lysine titers of 120–193.6 g L ${}^{-1}$ 11, 12.

In the recent years, *Vibrio natriegens* has emerged as a promising chassis strain for synthetic biology and biotechnological applications 13–15. Known as the fastest-growing non-pathogenic organism to date 13, 16, *V. natriegens* DSM759 holds the potential to replace traditional microbial workhorses in various industrial processes and laboratory environments. It is a facultative anaerobic, gram-negative, rod-shaped bacterium with a single polar flagellum 17. Ubiquitous in marine environments, this halophilic microorganism has adapted to its high salinity environment 18 and essentially relies on the presence of sodium ions for cell proliferation 19–21. While known toxins from the *Vibrio* genus are present in some *V. natriegens* strains 22, *V. natriegens* DSM759 meets biosafety level 1 standards, making it a suitable candidate for research and industrial applications. When cultivated in glucose-based mineral medium under aerobic conditions, the strain exhibits growth (1.48–1.7 h ${}^{-1}$ ) and glucose uptake rates (21.4–21.7 mmol g ${}^{-1}$ h ${}^{-1}$ ) 23, 24 which are about 2–3 times higher than observed for *E. coli*. Furthermore, *V. natriegens* is able to utilize over 60 different substrates as carbon and energy source 23, 25. Underscoring its metabolic flexibility, *V. natriegens* is capable of reducing alternative electron acceptors under oxygen-deprived conditions such as nitrate or Fe(III) citrate, and possesses the ability to fix atmospheric nitrogen 26. Its industrial potential has been demonstrated for the production of organic and amino acids (pyruvate 27, succinate 28 and alanine 23), of biopolymers and pigments such as polyhydroxybutyrate (PHB) 29, melanin 30, beta-carotene and violacein 31, for short-chain alcohols (2,3-butandiol 32 and 1,3-propandiol 33) and selenium nanoparticles 34.

Despite the industrial potential of *V. natriegens* DSM759, significant gaps remain in the understanding of its metabolism and regulatory networks. As an important step in further unravelling its unique biology, we aimed here at elucidating the genetic make-up and regulation of the biosynthetic pathways for L-lysine and related AFAAs formation. First, we constructed a pathway map for the AFAA biosynthesis in *V. natriegens* using computationally annotated genome sequence data from BioCyc and KEGG databases and compared it to the pathway design in *E. coli* and *C. glutamicum*. Next, we characterized the enzymatic activities encoded by homologous genes that are presumably involved in the L-lysine pathway and phenotyped deletion mutants to identify essential genes within the pathway. Finally, we investigated the enzymatic and transcriptional regulation of the L-lysine and related AFAAs biosynthesis pathways through enzymatic assays and transcriptome analysis, respectively. These insights provide a foundation for the development of *V. natriegens* into an overproducer of L-lysine and related AFAAs, further advancing its potential as a key organism in industrial biotechnology and synthetic biology.

## RESULTS AND DISCUSSION

### Identification of putative biosynthetic genes implicated in the L-lysine and related AFAAs pathways

**Figure 1:**
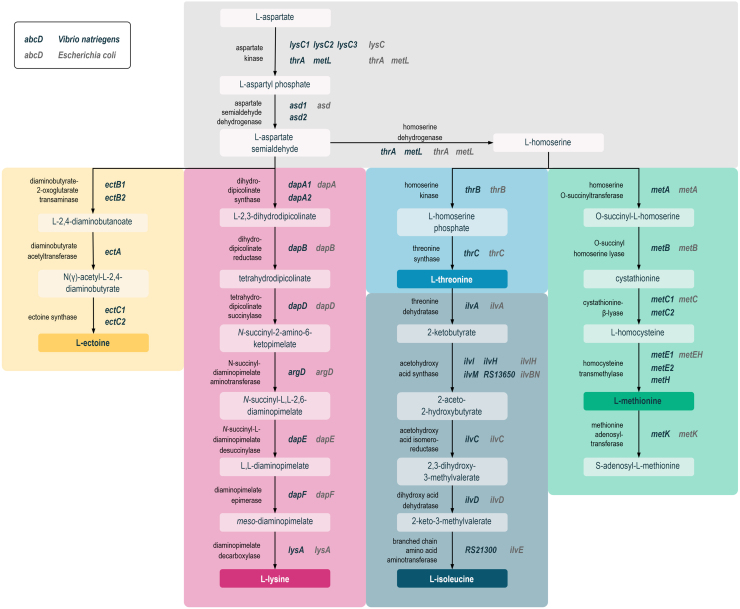
Pathway map of the L-lysine and related AFAAs biosynthesis pathways in *V. natriegens* and *E. coli*. Data was obtained from BioCyc 35 and KEGG databases 36. Genes are referred to by their gene symbol, if available, or by their locus tag in the format “*RS12345*” based on the RefSeq reference genome (BioSample ID SAMN03178087). In case of duplicate genes encoding putative isozymes, a consecutive numbering of the gene symbols was introduced.

To elucidate the genetic make-up of the L-lysine biosynthetic pathway in *V. natriegens*, we compiled automatically generated genome sequence annotation data provided by the BioCyc 35 and KEGG databases 36. Based on the compiled data, we constructed a comprehensive map of the biosynthetic network for L-lysine and related AFAAs assigning protein-encoding genes to all enzymatic reactions present within the analysed metabolic pathways (Figure 1). We then compared the design of the biosynthetic network in *V. natriegens* DSM759 to that in *E. coli* and *C. glutamicum*, whose corresponding metabolic routes are well studied owing to their industrial importance as amino acid overproducers.

It became evident that *V. natriegens* DSM759 employs only the succinylase sub-form of the diaminopimelic acid (DAP) pathway for L-lysine formation, exhibiting an overall identical pathway design to that of *E. coli*, whereas gram-positive *C. glutamicum* utilises both the succinylase and dehydrogenase variant of the DAP route 4, 6, 37. In addition to the four proteinogenic AFAAs produced by *E. coli* and *C. glutamicum* 3, 37, *V. natriegens* DSM759 synthesizes a fifth, non-canonical AFAA member – L-ectoine. L-ectoine is a compatible solute and chemical chaperone widely produced by halophilic or halotolerant *Bacteria* and some *Archaea* as a cytoprotectant against thermal and osmotic stress 38, 39. It is synthesized from L-aspartate via L-aspartate semialdehyde (ASA) in five consecutive enzymatic steps involving EctA, EctB and EctC encoded by the *ectABC* operon, which is evolutionarily conserved across Gram-negative and -positive bacteria 38, 39 including *V. natriegens* DSM759 (Supplementary data, Figure S1E). Another key difference between the analysed bacterial AFAA pathways is the presence of gene duplicates in *V. natriegens* DSM759. For several enzymatic reactions of the branched network, duplicate genes putatively encoding isozymes were identified on the first (Chr1, 3.24 Mbp) and second chromosome (Chr2, 1.92 Mbp). Regarding the L-lysine pathway, this includes three putative mono-functional aspartate kinase (AK), two putative aspartate semialdehyde dehydrogenase (ASD), and two putative dihydrodipicolinate synthase (DHDPS) encoding genes. To clearly denote these genes in the text, we introduced a consecutive numbering of the respective gene symbols, e.g. *Vn.lysC1, Vn.lysC2* and *Vn.lysC3* (Supplementary data, Table S2). Gene duplicates were also found within the pathways of the other AFAAs, e.g. *Vn.metC1/2*, *Vn.metE1/2* and *Vn.ectB1/2*. The presence of such gene duplications in the genome of *V. natriegens* DSM759 is not a novel observation, but rather common for members of the fast-replicating *Vibrionaceae* family harbouring two circular chromosomes 40. Automatically generated sequence annotations of the *V. natriegens* DSM759 genome revealed 4578 coding DNA sequences (CDS), 11 rRNA operons and 129 tRNA encoding genes. Lee *et al.,* 2019 identified 587 core (essential and growth-supporting) genes via functional genomics based on CRISPR interference (CRISPRi), with 96% of these genes being located on the larger Chr1. As the duplicates of core genes tend to be located on Chr2, it has been hypothesized that Chr2 functions as an “evolutionary test bed” in *Vibrio* species 41.

Consequently, the question arises as to whether these gene duplicates in fact encode functional enzymes and what physiological role they play. Analysing the local genomic context of the target genes can provide an initial indication of the relevance and reliability of the annotated gene functions. In case of the three putative mono-functional AK-encoding genes (*Vn.lysC1-3*) located on Chr1, only *Vn.lysC3* was found in a genomic context which corroborates its role as a functional AK. The gene is part of the L-ectoine biosynthesis operon *Vn.ectAB1C1_lysC3*, thus suggesting that Vn.LysC3 functions as an AK with a specific role in L-ectoine formation (Supplementary data, Figure S1A) 3, 38, 39. In contrast, *Vn.lysC1* and *Vn.lysC2* are not part of any operon, and while *Vn.lysC1* is located next to *Vn.metH1* of the L-methionine biosynthesis pathway, the neighbouring genes of *Vn.lysC2* do not have functions associated with the AFAA metabolism. Likewise, the two *asd* genes (*Vn.asd1/2*) located on Chr1, which putatively encode ASD enzymes, lack any informative gene context. The putative DHDPS-encoding gene *Vn.dapA1* (on Chr1) is adjacent to the same gene as its *E. coli* counterpart (*dapA-bamC*), suggesting that Vn.DapA1 functions as a DHDPS in *V. natriegens* DSM759. However, the genetic context of *Vn.dapA2* (on Chr2) does not provide sufficient information to infer its physiological role.

To answer the question of the physiological relevance of the putative isozymes within the L-lysine biosynthetic pathway and to verify the automatically generated annotations of the respective protein-encoding genes, we created single and multiple deletion mutants of the gene duplicates and characterised their growth phenotypes. Additionally, we cloned the target genes into pET28 vectors for protein production and subsequently analysed the activities of the purified enzymes.

### Phenotypic characterization of knockout strains targeting putative L-lysine and related AFAAs biosynthetic genes

**Figure 2:**
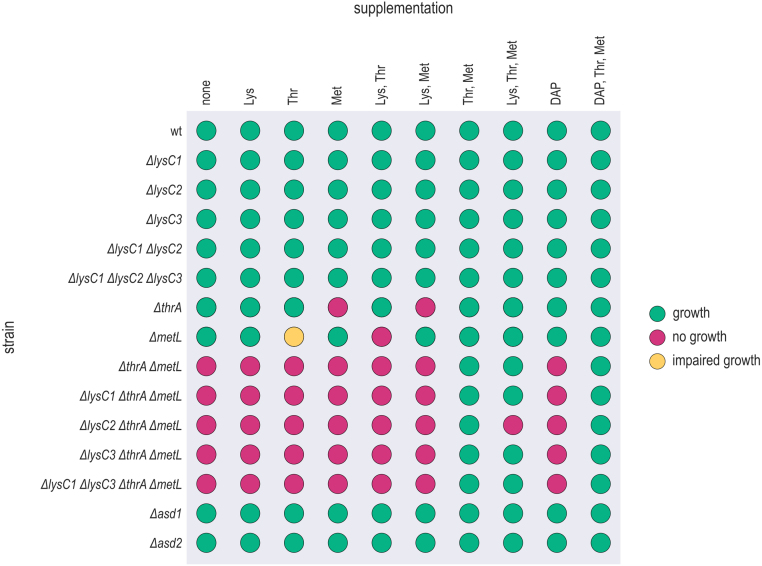
Phenotyping of L-lysine biosynthesis pathway gene deletion mutants. Putative AK, AK-HD and ASD isozymes encoding genes were deleted *via* homology recombination using a pDM4-derived vector system and candidate gene deletion mutants were spotted onto VN mineral media agar plates supplemented with 0.05 g L ${}^{-1}$ L-lysine (Lys), 0.2 g L ${}^{-1}$ L-threonine (Thr), 0.2 g L ${}^{-1}$ L-methionine (Met) and/or 0.25 g L ${}^{-1}$ DAP to elucidate function and essentiality of isozymes. Growth vs. non-growth behaviour was analysed.

Since the automatic genome annotation revealed gene duplicates putatively encoding isozymes for multiple enzymatic reactions of the L-lysine biosynthetic pathway, we analysed the physiological role and essentiality of those AK, bi-functional aspartate kinase/homoserine dehydrogenase (AK-HD) and ASD enzymes by targeted deletion of the corresponding coding genes. Gene deletion mutants were created *via* homology recombination using a pDM4-derived vector system. The strains were spotted onto VN mineral media agar plates supplemented with L-lysine, L-threonine, L-methionine and/or DAP for assessing their growth behaviour (Figure 2; Supplementary Data: Figure S3).

All single mutants were able to grow on non-supplemented mineral medium, revealing redundancy of all deleted enzymatic activities. To analyse the physiological role of the three putative mono-functional AK isozymes, firstly, the growth behaviour of single, double and triple Δ *Vn.lysC1-3* knockout strains was characterized. All strains carrying deletions of *Vn.lysC1-3* were able to grow on non-supplemented minimal medium as well as under all nine supplementation conditions (Figure 2). Therefore, no final conclusion about the physiological role of the *Vn.lysC* genes could be inferred. However, these results indicate that the AK activity is (also) encoded elsewhere on the *V. natriegens* genome, and that it is presumably provided by the putative bi-functional AK-HD enzymes – Vn.ThrA and/or Vn.MetL. To further investigate this question, single and double mutants of the putative bi-functional AK-HD encoding genes were created (*V. natriegens* Δ *thrA*, Δ *metL,* Δ *thrA* Δ *metL*) and their growth behaviour characterized. While both single mutants were able to grow under non-supplemented conditions, growth of the double mutant was only observed when both L-threonine and L-methionine were present (Figure 2). These results indicate that *Vn.thrA* and *Vn.metL* are the sole HD encoding genes in *V. natriegens* DSM759 and that both enzymes can mutually complement their functions as no L-threonine/L-methionine-auxotrophy was observed for the single mutants. To further analyse the physiological roles of the three *Vn.lysC* genes, we aimed at creating triple, quadruple and quintuple mutants by combining *lysC1-3* gene deletions with the Δ *thrA* Δ *metL* background. *V. natriegens* Δ *lysC1* Δ *thrA* Δ *metL, V. natriegens* Δ *lysC2* Δ *thrA* Δ *metL, V. natriegens* Δ *lysC3* Δ *thrA* Δ *metL* and *V. natriegens* Δ *lysC1* Δ *lysC3* Δ *thrA* Δ *metL* were successfully constructed. As expected, the strains were only able to grow in the presence of both L-threonine and L-methionine (Figure 2), and did not require DAP supplementation for growth. However, despite extensive efforts, we were unable to generate neither the quadruple *V. natriegens* Δ *lysC1* Δ *lysC2* Δ *thrA* Δ *metL* mutant nor the quintuple *V. natriegens* Δ *lysC1* Δ *lysC2* Δ *lysC3* Δ *thrA* Δ *metL* mutant. In particular, two strategies were tested for the chromosomal gene deletion: the previously applied homologous recombination based technique using a pDM4-derived vector system and, additionally, NT-CRISPR 42, 43. To account for the predicted DAP-auxotrophy of the target mutant strains, cultures were supplemented with DAP alone as well as with DAP and all AFAAs of the branched pathway (L-lysine, L-threonine, L-methionine and L-ectoine). Despite these interventions, neither strategy resulted in the successful construction of the desired strains. This outcome points to an inherent biological constraint rather than a technical limitation. Overall, the results indicate functional redundancy of the *Vn.lysC*-encoded AK activities; however, due to our failing attempts to construct strains devoid of all potential AK-encoding genes (*V. natriegens* Δ *lysC1* Δ *lysC2* Δ *thrA* Δ *metL* and *V. natriegens* Δ *lysC1* Δ *lysC2* Δ *lysC3* Δ *thrA* Δ *metL),* verification of their function based on mutant phenotypes remains inconclusive and requires direct enzymatic assays (see below). Finally, we aimed to create single deletion mutants of *Vn.asd1* and *Vn.asd2*, as well as the double mutant (*V. natriegens* Δ *asd1* Δ *asd2*). The single knockout strains were successfully generated and were able to grow on non-supplemented mineral medium as well as under all supplementation conditions (Figure 2), thus, not showing any DAP-auxotrophic behaviour. These results suggest that both *Vn.asd1* and *Vn.asd2* encode functional ASD enzymes which participate in the AFAAs biosynthesis pathway. In contrast, construction of the double *Vn.asd1/2* mutant was unsuccessful, likely due to the same biological constraint that prevented generation of strains lacking all putative AK-encoding genes. One possible explanation is that *V. natriegens* may be unable to import DAP, which would prevent external DAP from compensating for the loss of endogenous DAP biosynthesis in strains deleted for all AK or ASD encoding genes.

In addition to identifying the structural genes encoding enzymes involved in the AFAA metabolism, analysis of the growth behaviour of the knockout mutants may also provide insight into putative regulatory mechanism affecting the enzymes participating in this biosynthetic network. In particular, the Δ *Vn.thrA* and Δ *Vn.metL* mutants failed to grow on media supplemented with L-methionine and with L-lysine plus L-threonine, respectively. Furthermore, the *V. natriegens* Δ *lysC2* Δ *thrA* Δ *metL* strain did not grow when L-lysine, L-threonine, and L-methionine were supplemented simultaneously, whereas growth was maintained when only L-threonine and L-methionine were present (Figure 2). Although the agar plate spotting assay was intended only to assess whether a mutant could grow under a given condition or not, we observed a markedly delayed onset of growth for the Δ *Vn.metL* strain when cultivated in the presence of L-threonine. Therefore, growth behaviour of this strain was further examined in liquid culture (Supplementary Data, Figure S2). Consistent with the plate-based assay, Δ *Vn.metL* exhibited robust growth under most conditions (growth rates of 1.2–1.4 h ${}^{-1}$ ) but failed to grow under simultaneous L-lysine plus L-threonine supplementation. However, in liquid culture, the delayed growth onset became quantifiable: the Δ *Vn.metL* strain displayed a markedly reduced growth rate of 0.09 ± 0.01 h ${}^{-1}$ (corresponding to an approximately 93% reduction compared to the non-supplemented condition) when L-threonine was provided as the sole amino acid supplement. Together, these observations are consistent with potential regulatory effects of the tested amino acids on the *in vivo* activities of Vn.LysC1/3, Vn.ThrA and Vn.MetL. Specifically, the activity of Vn.MetL may be reduced by L-methionine, leading to growth restriction of Δ *Vn.thrA* due to limited L-threonine biosynthesis. Likewise, the activity of Vn.ThrA may be reduced by L-threonine (and L-lysine), resulting in limited L-methionine availability and consequently restricted growth of Δ *Vn.metL*. Finally, the presence of L-lysine may reduce the activity of Vn.LysC1 and/or Vn.LysC3, resulting in the absence of any active AK in the *V. natriegens* Δ *lysC2* Δ *thrA* Δ *metL* strain and consequently limiting DAP biosynthesis, which could underlie the observed growth arrest. It is not a novel observation that AFAAs regulate their own biosynthetic pathways. A common feature of this regulation is feed-back inhibition targeting the first enzymatic step within a pathway, thereby controlling the overall flux of the biosynthetic route. For instance, in *E. coli*, the expression of bi-functional AK-HD encoding genes – *Ec.thrA* and *Ec.metL*, is repressed by L-threonine/L-isoleucine and L-methionine, respectively. Additionally, Ec.ThrA is allosterically inhibited by L-threonine and the mono-funtional Ec.LysC is affected by L-lysine on both the enzymatic and transcriptional level 44. In *V. natriegens* DSM759 a similar regulatory pattern appears to exist. However, it remains to be verified if Vn.LysC1/3, Vn.ThrA and Vn.MetL are indeed sensitive to amino acid-mediated inhibition, and whether such effects result from direct allosteric regulation of the enzymes and/or from transcriptional repression of the corresponding genes. To address this, regulation of these enzymes and of the AFAA gene network was investigated at both levels *via* enzymatic assays and transcriptome analysis, respectively.

### Verification of the putative enzymatic activities of the L-lysine biosynthesis pathway and characterization of their regulation

To further elucidate the physiological roles of the isozymes, activities of putative AK, AK-HD, ASD and DHDPS enzymes were verified via enzymatic assays using the purified proteins. Additionally, the regulation of these enzymes was investigated by measuring their *in vitro* activities in presence of the end-products of the branched AFAA pathways (i.e. L-lysine, L-threonine, L-isoleucine, L-methionine and L-ectoine).

AK activity was confirmed for Vn.LysC1 and Vn.LysC2 (Table 3), supporting the annotation of the corresponding genes and indicating the presence of two catalytically active mono-functional AKs in *V. natriegens* DSM759. These results are also consistent with our previous findings from the growth assays of knockout strains, which indicated functional redundancy of the *Vn.lysC*-encoded AK activities, as the Δ *Vn.lysC1* Δ *Vn.thrA* Δ *Vn.metL,* Δ *Vn.lysC2* Δ *Vn.thrA* Δ *Vn.metL* and Δ *Vn.lysC3* Δ *Vn.thrA* Δ *Vn.metL* mutant strain did not exhibit a DAP auxotrophic phenotype (Figure 2). In contrast, no activity was detected for Vn.LysC3 and therefore no definitive conclusion can be drawn regarding the physiological role of this putative AK encoded on the *ect* operon. For the putative bi-functional AK-HD enzymes (Vn.ThrA and Vn.MetL), both the AK as well as the HD activity was demonstrated (Table 3). Together with the growth phenotypes of the corresponding gene deletion mutants (Figure 2), this confirms the correct annotation of the encoding genes. The kinetic parameters of Vn.LysC1, Vn.LysC2, Vn.ThrA and Vn.MetL are presented in Table 3. They are largely comparable to those of other microbial AK/AK-HD enzymes 44–47.

Subsequently, the inhibition profiles of the confirmed AK enzymes were analysed in response to the presence of AFAAs (L-lysine, L-threonine, L-isoleucine, L-methionine and L-ectoine). The individual addition of L-threonine, L-isoleucine, L-methionine or L-ectoine to the *in vitro* assays had no significant influence on the enzymatic activity of either Vn.LysC1/2 enzyme (Table 3, Supplementary Data: Figure S4-A). However, the addition of L-lysine led to a strong reduction in Vn.LysC1-dependent AK activity (5% residual activity at 5 mM L-lysine), while no inhibitory effect was observed for Vn.LysC2 (103% activity). For the bi-functional AK-HD enzymes, no inhibition of Vn.MetL was observed under any tested amino acid supplementation, whereas Vn.ThrA activity was slightly reduced by L-threonine, retaining about 80% residual activity (Table 3, Supplementary Data Figure S4B). The mechanisms and structural requirements of allosteric inhibition of AK enzymes have been well described, especially in *E. coli* and *C. glutamicum* 48–50. These enzymes comprise a catalytic AK domain and a regulatory domain containing two or four ACT motifs. In the case of bi-functional AK-HD enzymes, they are fused to a catalytic HD domain. Allosteric inhibitors bind to the ACT domains and trigger conformational changes of both the regulatory and the catalytic AK domain, thereby hindering catalysis 51. Hence, alignments of the AK/AK-HD sequences may identify conserved domains and provide a structural explanation for the observed differences in amino acid-mediated inhibition (Supplementary Data, Figure S5). In Vn.LysC1, ACT domains similar to those found in Ec.LysC were identified. Together with its similarly strong L-lysine sensitivity, this supports the hypothesis that L-lysine inhibits Vn.LysC1 through a regulatory mechanism analogous to that of Ec.LysC. In contrast, Vn.LysC2 lacks ACT domains, providing structural support for its insensitivity to the tested amino acids. Likewise, the absence of ACT domains in both Ec.MetL and Vn.MetL is consistent with their lack of inhibition by any of the supplemented amino acids. Therefore, the growth restriction observed for the Δ *Vn.thrA* mutant strain upon L-methionine supplementation (Figure 2) cannot be explained at the enzymatic level, suggesting that the underlying regulatory mechanism may instead occur at the transcriptional level (see below). On the other hand, both Ec.ThrA and Vn.ThrA contain ACT domains, which should, in principle, render them sensitive to allosteric feedback inhibition. However, strong L-threonine-mediated inhibition was observed only for Ec.ThrA, as previously reported 44, whereas Vn.ThrA was only slightly affected by L-threonine. Given their structural similarities, this difference is somewhat unexpected. Nevertheless, previous studies have shown that several AK/AK-HD enzymes are inhibited by multiple amino acids acting in a concerted manner. For example, the sole AK of *C. glutamicum* is inhibited in a concerted manner by L-lysine and L-threonine, while that of *Arabidopsis thaliana* is jointly inhibited by L-lysine and S-adenosyl methionine (SAM) 49, 50, 52. Consistent with this, the stronger growth restriction of the Δ *Vn.metL* mutant strain upon combined L-lysine and L-threonine supplementation – compared to L-threonine alone – suggests that simultaneous presence of both amino acids may be required for significant inhibition of the Vn.ThrA enzyme. However, *in vitro* assays demonstrated that concurrent addition of L-lysine and L-threonine, or a mixture of L-lysine, L-threonine, L-isoleucine, and L-methionine (Supplementary Data: Figure S4-C), did not further reduce the activity of either Ec.ThrA or Vn.ThrA compared with L-threonine supplementation alone. Moreover, sequence alignment revealed that Ec.ThrA and Vn.ThrA share conserved residues at positions 345 and 433, where mutations in Ec.ThrA have been associated with L-threonine feedback resistance 53, 54. Consequently, the basis for the observed difference in L-threonine sensitivity of Ec.ThrA and Vn.ThrA remains unclear.

Consistent with ability of the Δ *Vn.asd1* and Δ *Vn.asd2* single-knockout mutants to grow without any supplementation, ASD activity was demonstrated for both Vn.Asd isozymes (Table 3). These results further support the hypothesis that both Vn.Asd1 as well as Vn.Asd2 participate in the AFAAs biosynthesis pathway. Vn.Asd1 exhibited an about 9-fold higher activity on L-aspartyl phosphate than Vn.Asd2. A similar behaviour was previously observed for the two ASD enzymes encoded on the *V. cholerae* genome (Vc.Asd1 and Vc.Asd2), where Vc.Asd1 was about two times more active than its isozyme 55.

DHDPS activity was confirmed for both putative Vn.DapA isozymes of *V. natriegens* (Table 3). The kinetic parameters of Vn.DapA1 were in a similar range as reported for Ec.DapA. In contrast, the activity of Vn.DapA2 was hardly measurable (0.07 ± 0.01 U mg ${}^{-1}$ ) and no saturation was observed under the applied conditions (max. 1 mM ASA), indicating that Vn.DapA1 is the major DHDPS in *V. natriegens* DSM759. In *E. coli*, DHDPS is allosterically inhibited by L-lysine 56. Here, we show that the addition of L-lysine also resulted in a strong decrease of Vn.DapA1-dependent DHDPS activity (5% residual activity at 5 mM L-lysine) indicating allosteric inhibition of Vn.DapA1 through L-lysine, whereas Vn.DapA2 was only slightly affected, retaining 83% residual activity (Supplementary Data: Figure S4-D).

In summary, we demonstrated that L-lysine biosynthesis in *V. natriegens* DSM759 is regulated at the enzymatic level through end-product feedback inhibition of the mono-functional AK (Vn.LysC1) and the DHDPS (Vn.DapA1) (Figure 5). This regulatory pattern is identical to the one described for the analogous *E. coli* pathway. However, unlike the metabolic setup in *E. coli*, the *V. natriegens* DSM759 genome encodes an additional mono-functional AK (Vn.LysC2) which is L-lysine-insensitive as well as an additional DHDPS isozyme (Vn.DapA2) which is only slightly inhibited by L-lysine.

### Transcriptional regulation of the biosynthesis of L-lysine and related AFAAs

We next set out to study the transcriptional response of *V. natriegens* to the presence of AFAAs. To this end, the *V. natriegens* DSM759 Δ *dns* reference strain was cultivated in VN mineral medium which was supplemented with either L-lysine, L-threonine, L-isoleucine, L-methionine, or L-ectoine (20 mM each). Total RNA was isolated from exponentially growing cells (OD ${}_{\mathrm{600 nm}}$ of approx. 0.5 at harvest). The presence of the AFAAs in the cultivation medium at the time of cell harvest was verified by HPLC analysis (not shown). mRNA was enriched by a ribosomal RNA depletion protocol and subsequently sequenced. Reads were mapped to the RefSeq GCF_001456255.1 reference genome, read counts were normalised and statistically analysed to identify differentially expressed genes following the procedure depicted in Figure S6.

An initial principal component analysis (PCA) revealed that replicates of the different supplementation conditions formed distinct clusters that were clearly distinguishable from the reference condition. The only exception to this trend was observed for the L-ectoine-supplemented samples, which were indistinguishable from the reference samples (Supplementary data, Figure S7). Indeed, a more detailed statistical analysis revealed that no differential gene expression occurred in the L-ectoine-treated cultures (Supplementary data, Figure S8). This observation may be ascribed to poor uptake of the amino acid in the absence of osmotic stress, as it was suggested previously for L-ectoine import in *V. parahaemolyticus* 57.

**Figure 3:**
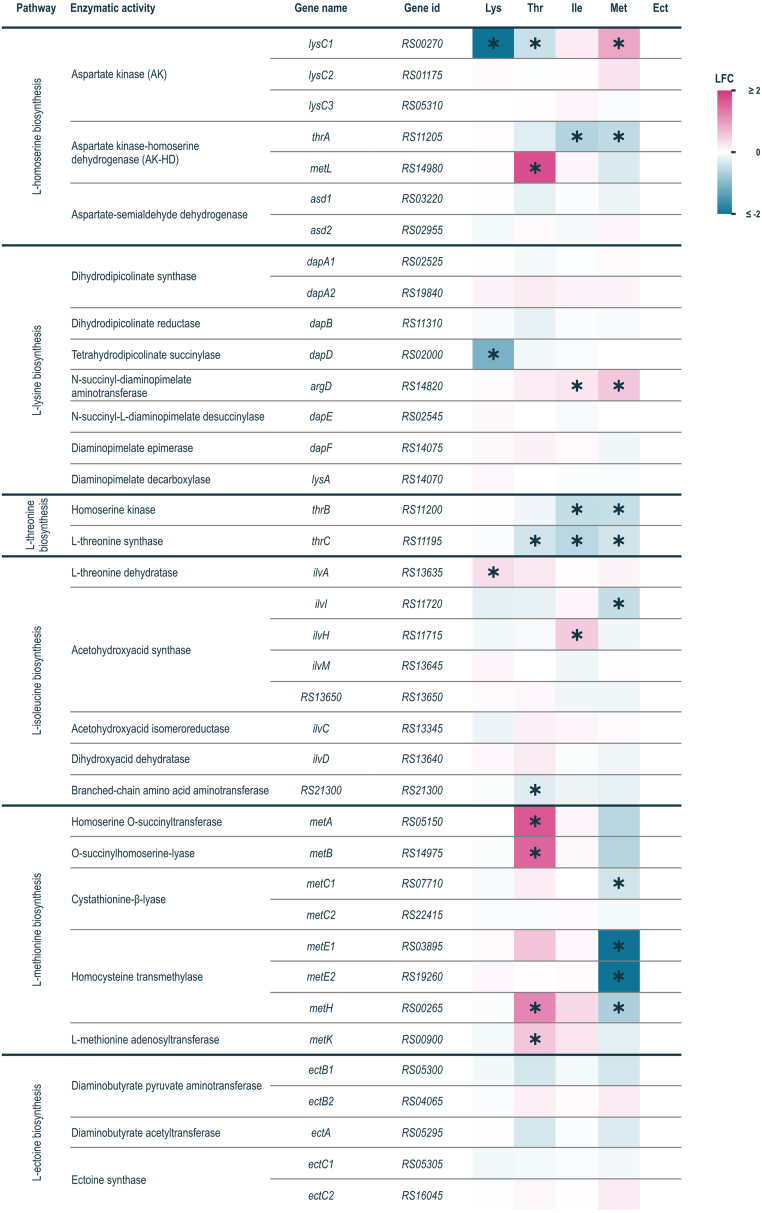
Expression levels of the genes implicated in the L-lysine and related AFAAs biosynthesis pathways in *V. natriegens*. Colour indicates strength of the log2 fold change (LFC) of gene expression levels for each supplementation condition (L-lysine (Lys), L-threonine (Thr), L-isoleucine (Ile), L-methionine (Met) and L-ectoine (Ect)) compared to the reference condition (no supplement) (^⁎^ – differentially expressed genes (DEGs)).

**Figure 4:**
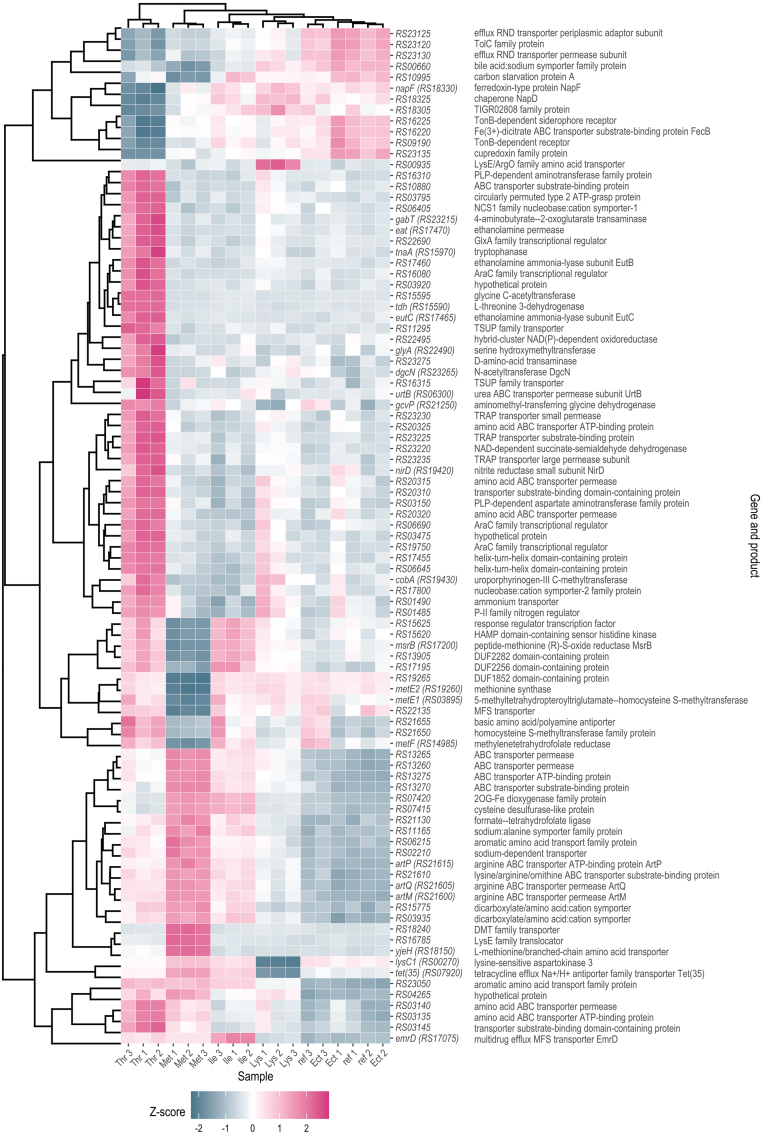
Hierarchical clustering of differentially expressed genes. Heat map of genes that meet the criteria p ${}_{\mathrm{adj}}$ < 0.05 and |LFC| ≥ 2 in at least one supplementation condition (L-lysine (Lys), L-threonine (Thr), L-isoleucine (Ile), L-methionine (Met) and L-ectoine (Ect)). For each gene (row), the rlog-transformed counts across all samples (columns) were z-transformed and subsequently clustered hierarchically based on their Euclidean distance. Rows are labelled with the corresponding gene name and gene product.

Our analysis on the transcriptional response to the different AFAAs focused first on their biosynthetic pathways (Figure 3) and was then extended to the genome-wide transcriptional response triggered by the presence of elevated concentrations of the individual AFAAs (Figure 4). In general, presence of L-lysine, L-threonine, L-isoleucine and L-methionine resulted in transcriptional repression of their corresponding biosynthetic pathways, as it was reported before for *E. coli* and *C. glutamicum* 4–6. The AK reaction is the entry point into the AFAA pathways and was found to be a hotspot of transcriptional regulation (Figure 3): while L-lysine and L-threonine caused statistically significant repression of the mono-functional AK encoding gene *Vn.lysC1* (LFC = −3.96 and LFC = −0.44), L-isoleucine and L-methionine significantly repressed the bi-functional AK-HD encoding gene *Vn.thrA* (LFC = −0.63 and LFC = −0.52). Additionally, *Vn.lysC1* and the second bi-functional AK-HD encoding gene *Vn.metL* were differentially upregulated under L-methionine (LFC = 0.88) or L-threonine (LFC = 1.75) supplementation, respectively. Interestingly, the experimentally confirmed mono-functional AK encoding gene *Vn.lysC2* and the elusive *Vn.lysC3* gene both escaped transcriptional regulation by any of the tested amino acids. However, it should be noted that for *Vn.lysC3*, as well as for the L-ectoine biosynthesis genes, *Vn.ectAB1C1*, low absolute read counts were measured (less than 10 TPM; see Supplementary data, Figure S9) suggesting repression of the whole *Vn.ectAB1C1_lysC3* operon under all tested amino acids supplementation conditions. Given the physiological role of L-ectoine as a compatible solute whose biosynthesis is typically induced under osmotic stress 38, 46, 58, we hypothesize that higher NaCl concentrations would lead to induction of *Vn.ectAB1C1_lysC3* expression. Indeed, analysis of a previously published dataset by Shin *et al., 2024* 59 supports this assumption, showing upregulation of the *Vn.ectAB1C1_lysC3* operon at elevated NaCl concentrations (500 mM and 800 mM compared to 200 mM; p ${}_{\mathrm{adj}}$ < 1.1e ${}^{-83}$ , LFC > 6, n = 2; Supplementary Data: Figure S10, Table S7).

**Figure 5:**
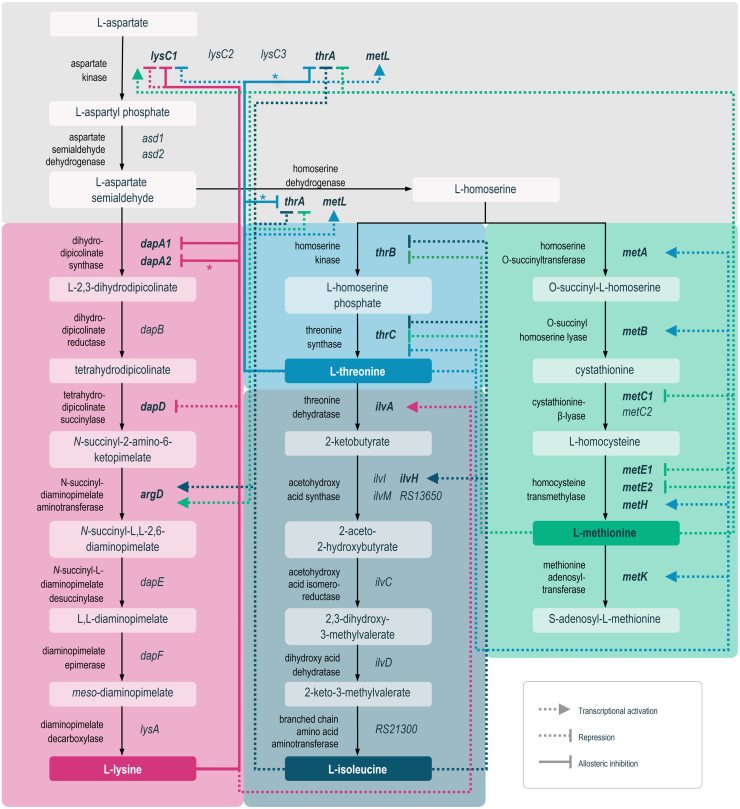
Summary of observed transcriptional and enzymatic regulation of the L-lysine and related AFAAs biosynthesis pathways in *V. natriegens*. Transcriptional activation (dashed line, arrows) and repression (dashed line, T-bar) as well as product feed-back inhibition on the enzymatic level (solid line, T-bar) are shown. The line colour indicates the respective amino acid causing the observed regulation event. Asterisks (^⁎^) indicate cases where the enzyme was only slightly inhibited, retaining approximately 80% of its activity.

The transcriptional response of the AFAA network to the presence of L-lysine was very focused and essentially limited to differential downregulation of two L-lysine pathway genes, *Vn.lysC1* (LFC = −3.96) and *Vn.dapD* (LFC = −1.11). In *E. coli*, *Ec.lysC* expression is governed by a L-lysine-sensitive dual riboswitch that regulates translation initiation, transcription termination and mRNA decay 60. Riboswitches are RNA elements located in 5’ untranslated regions (UTR) of some mRNAs. They consist of an aptamer domain which is highly conserved in both sequence and structure and allows for specific metabolites to bind and control gene expression on the translational or transcriptional level. L-lysine-specific riboswitches, also named LYS element or L box, were identified across different members of γ -Proteobacteria and the *Bacillus/Clostridium* genus, where they are located in 5’UTR regions of multiple genes involved in L-lysine biosynthesis and transport 61, 62. Here, a multiple sequence alignment (MSA) comparing the 5’UTR of *Vn.lysC1* to the known *lysC* riboswitches of *E. coli*, *B. subtilis*, *V. cholerae*, *V. fischeri*, *V. vulnificus* and *V. parahaemolyticus* 61 revealed a high RNA sequence similarity between the analysed regions (Supplementary data, Figure S12). Furthermore, prediction of the RNA secondary structure of the *Vn.lysC1* 5’UTR matched the conserved structure of the LYS element reported in literature 61, 62 indicating that *Vn.lysC1* expression may be governed by a L-lysine-sensitive riboswitch. Additionally, in *E. coli*, transcription initiation of *Ec.dapD,* together with *Ec.lysC, Ec.asd, Ec.dapB, Ec.lysA* and the L-lysine transporter encoding gene *Ec.lysP,* is stimulated by the transcriptional regulator ArgP 63. Binding of L-lysine to ArgP, prevents activation of these genes. Likewise, this may be the cause for the here observed downregulation of *Vn.dapD* in presence of L-lysine.

In contrast, L-threonine, L-isoleucine and L-methionine affected the expression of a larger set of genes. Specifically, L-threonine and L-isoleucine caused repression of the entire L-threonine biosynthetic pathway, comprising of *Vn.thrA* (LFC ${}_{\mathrm{Thr}}$ = −0.25 and LFC ${}_{\mathrm{Ile}}$ = −0.63), *Vn.thrB* (LFC ${}_{\mathrm{Thr}}$ = −0.12 and LFC ${}_{\mathrm{Ile}}$ = −0.49) and *Vn.thrC* (LFC ${}_{\mathrm{Thr}}$ = −0.38 and LFC ${}_{\mathrm{Ile}}$ = −0.57), albeit downregulation of *Vn.thrA* and *Vn.thrB* expression was not statistically significant in the case of L-threonine supplementation. In *E. coli*, *Ec.thrABC* genes are also repressed by L-threonine and L-isoleucine. The effect is mediated by ribosome-mediated transcriptional attenuation 64, 65. Transcriptional attenuation is based on a special leader sequence upstream of the structural gene which encodes a short peptide consisting of several codons of the controlling amino acid(s) and a region participating in forming a stem and loop secondary RNA structure. When sufficient respective amino acyl tRNAs are available, the ribosome can read through the leader sequence leading to the formation of a terminator hairpin structure, thus pre-terminating the transcription of the structural gene. When, on the other hand, cognate tRNAs are lacking, translation of the short peptide stalls. This pause enables the formation of an anti-terminator structure, which allows transcription of the biosynthetic genes. In *E. coli*, the *Ec.thr* leader sequence (*Ec.thrL*) is located 147 bp upstream of *Ec.thrA* and contains a total of eight L-threonine as well as four L-isoleucine codons. Here, sequence analysis revealed that *V. natriegens* possesses a similar open reading frame (54 bp/18 AA long) 163 bp upstream of the *Vn.thrABC* coding sequence (2,417,213–2,417,160) including five L-threonine and five L-isoleucine codons (Supplementary data, Figure S13). Furthermore, a hairpin containing a poly-U motif, resembling the *E. coli* terminator structure, was identified within the *V. natriegens thr* leader sequence. Together, these observations suggest that the reduced expression of the *Vn.thrABC* operon in the presence of L-threonine and L-isoleucine may be controlled by ribosome-mediated transcriptional attenuation in *V. natriegens* DSM759 in a manner similar to that observed in *E. coli*.

The presence of L-methionine triggered transcriptional downregulation of almost all genes implicated in the L-methionine biosynthetic pathway, i.e. *Vn.metA* (LFC = −0.59), the *Vn.metBL* operon (LFC = −0.61 and −0.30), *Vn.metC1* (LFC = −0.39), *Vn.metE1/2* (LFC = −5.20 and −7.10), *Vn.metH* (LFC = −0.68) and *Vn.metK* (LFC = −0.23) (statistical significance not given for all cases, Figure 3). In *E. coli*, the repression of all *Ec.met* genes, except *Ec.metH*, is attributed to the action of the repressor MetJ and its L-methionine-derived corepressor SAM. *Ec.metH* is indirectly regulated by SAM-MetJ, which in turn represses transactivator MetR of *Ec.metH* and *Ec.metE* transcription. SAM-MetJ binds to specific regions of the *met* gene promoters, termed *met* boxes. *Via* DNase foot printing and sequence analysis, an eight-base consensus sequence “AGACGTCT” of the *met* boxes was identified, with *E. coli met* genes generally having two to five contiguous *met* boxes 66–68. Here, through an iterative search of 5’ sequences of *V. natriegens met* genes, we identified multiple contiguous *met* boxes upstream of *Vn.metA, Vn.metB, Vn.metC1/2, Vn.metE1/2* as well as *Vn.metH* (Supplementary data, Figure S14) indicating that the *met* genes of *V. natriegens* DSM759 are likely to be governed by SAM-MetJ-mediated regulation. Furthermore, transcriptional repression of *Vn.metL* in response to L-methionine may provide an explanation for the growth defect of the *V. natriegens* Δ *thrA* mutant under L-methionine supplementation (Figure 2). This phenotype could not be explained for by enzyme-level regulation, which suggests that transcriptional control, rather than allosteric inhibition, primarily modulates the metabolic flux under these conditions. The ensuing restricted biosynthesis of L-threonine could, therefore, account for the growth arrest of the Δ *Vn.thrA* strain.

Interestingly, beyond the expected transcriptional downregulation of AFAA pathways in response to their specific metabolic end-products, our results indicate a bidirectional regulatory crosstalk between the L-threonine and L-methionine biosynthetic pathways in *V. natriegens*DSM759. L-threonine supplementation triggered induction of L-methionine biosynthetic genes (LFC ${}_{metA}$ = 1.67, LFC ${}_{metB}$ = 1.53, LFC ${}_{metE1}$ = 0.60, LFC ${}_{metH}$ *=* 1.20, LFC ${}_{metK}$ *=* 0.57), whereas L-methionine led to repression of genes involved in L-threonine biosynthesis (LFC ${}_{thrA}$ = −0.52, LFC ${}_{thrB}$ = −0.47, LFC ${}_{thrC}$ = −0.39, Figure 3). While the physiological significance remains unclear, these transcriptional data suggest that strategies to enhance L-threonine production in *V. natriegens* DSM759 should take into account a possible concurrent activation of the L-methionine pathway.

After having studied the transcriptional regulation of the specific AFAA biosynthetic pathways, we widened the scope of the analysis and characterized the transcriptional response of *V. natriegens* DSM759 to the presence of these amino acids on a genome-wide level (Figure 4). To this end, the dataset was filtered for genes showing an absolute LFC ≥ 2 associated with a Benjamini-Hochberg-adjusted p-value < 0.05 in at least one of the tested conditions. We found that 4, 7, 37, or 60 genes were differentially expressed in presence of L-lysine, L-isoleucine, L-methionine or L-threonine, respectively, when compared to the reference condition (Figure 4, Supplementary Data: Figure S8, Table S3). These numbers indicate an extremely focussed transcriptional response to L-lysine and L-isoleucine supplementation. Interestingly, the presence of L-lysine caused differential upregulation of *RS00935* (LFC = 3.38, p ${}_{\mathrm{adj}}$ = 2.2 × 10 ${}^{-46}$ ), putatively encoding a homologue to the L-lysine exporter LysE of *C. glutamicum* 69. On the other hand, putative homologues of the *E. coli* L-lysine symporter *Ec.lysP* 70 and exporter *Ec.lysO* genes 71, *RS21655* (LFC = 0.00, p ${}_{\mathrm{adj}}$ = 0.97) and *RS07480* (LFC = 0.25, p ${}_{\mathrm{adj}}$ = 0.22), respectively, did not exhibit differential expression in any of the supplementation conditions tested (not shown). Gene set enrichment analysis was performed using Gene Ontology (GO) terms and KEGG pathway classifications (Supplementary Data, Figure S11, Table S4 and Table S5). GO-term analysis did not reveal any pronounced patterns beyond the expected impact on amino acid and nitrogen metabolism in response to the amino acid supplementation. In contrast, KEGG pathway analysis indicated that the presence of L-threonine triggers transcriptional changes in glycine, serine and threonine metabolism (vna00260, normalized enrichment score (NES) = 2.10, p ${}_{\mathrm{adj}}$ = 3.6 × 10 ${}^{-5}$ ), suggesting a metabolic reprogramming that facilitates their biosynthesis *via* L-threonine degradation. L-methionine supplementation, by comparison, resulted in repression of cysteine (vna00270, NES = −1.95, p ${}_{\mathrm{adj}}$ = 1.20 × 10 ${}^{-3}$ ), and sulphur metabolism (vna00920, NES = −2.19, p ${}_{\mathrm{adj}}$ = 1.58 × 10 ${}^{-4}$ ), which is consistent with the role of the cysteine-dependent transsulfuration pathway as a major route for L-methionine biosynthesis 72.

## CONCLUSION

This study provides a comprehensive analysis of the genetic organization and regulatory mechanisms governing the biosynthesis pathways of L-lysine and related AFAAs in *V. natriegens* DSM759. All work was performed using our laboratory strain, derived from the DSM759 Δ *dns* type strain 73, with its genome sequence verified (Supplementary Data, Table S6). We showed that the overall structure of the branched AFAA biosynthetic network closely resembles that of *E. coli*, with L-lysine being synthesized *via* the succinylase sub-form of the DAP route. Based on automatically generated genome annotation data, we were able to assign protein-encoding genes to all enzymatic reactions present within the analysed metabolic routes and create a complete pathway map. A distinctive feature of the AFAA biosynthetic network in *V. natriegens* DSM759, in contrast to established L-lysine and AFAA producers such as *E. coli* and *C. glutamicum*, is the presence of gene duplicates putatively encoding isozymes. We were able to experimentally confirm the enzymatic activity of most of these isozymes of the L-lysine biosynthetic route. Additionally, growth phenotyping of the corresponding gene deletion mutants revealed redundancy of all the deleted enzymatic activities, further highlighting the physiological relevance of the encoded isozymes. Next, we analysed the regulation mechanisms governing the AFAA pathways in *V. natriegens* DSM759 via enzymatic assays and RNA sequencing experiments. We showed that L-lysine exerts regulatory control over its own biosynthetic pathway at both the enzymatic and transcriptional levels, reflecting regulation patterns previously reported for *E. coli*. Specifically, L-lysine was shown to strongly inhibit the activity of two key enzymes – mono-functional AK (Vn.LysC1) and DHDPS (Vn.DapA1), as well as significantly repressing *Vn.lysC1* and *Vn.dapD* transcription. Likewise, L-threonine, L-isoleucine, and L-methionine were found to transcriptionally downregulate the expression of key genes within their respective metabolic pathways, also governing their own biosynthesis. In contrast, L-ectoine supplementation did not affect any transcriptional changes.

Finally, a particularly intriguing finding is the identification of an additional mono-functional AK Vn.LysC2, whose enzymatic activity and gene expression escaped any regulatory mechanism at both the enzymatic and transcriptional levels. This inherent feedback resistance distinguishes Vn.LysC2 from its counterparts and could provide a distinct advantage for the metabolic engineering of *V. natriegens* DSM759. In both *E. coli* and *C. glutamicum*, the AK reaction is considered a rate-limiting step in L-lysine biosynthesis, and obtaining feedback-insensitive LysC variants is a key challenge in strain development 47, 74–76. Although feedback-resistant *LysC* mutants have been generated, they are often accompanied by reduced catalytic activity – e.g., the L-lysine-resistant Ec.LysC:E250K retains only about 50% of the wild-type activity 76. By comparison, Vn.LysC2 displayed a v ${}_{\max}$ similar to Ec.LysC, but about half that of its *V. natriegens’* homolog Vn.LysC1, with K ${}_{M}$ values similar to those of Vn.LysC1. Together, the kinetic properties of Vn.LysC2 and its intrinsic feedback resistance highlight its potential as a promising candidate for engineering L-lysine-producing *V. natriegens* DSM759 strains.

In summary, the regulatory insights obtained in this work establish a strong foundation for the rational engineering of *V. natriegens* DSM759 as a production host for L-lysine and other AFAAs, further advancing its potential as a chassis organism in synthetic biology and industrial biotechnology.

## MATERIALS AND METHODS

### Reagents and chemicals

Chemicals, solvents and oligonucleotides were purchased from Sigma-Aldrich (Darmstadt, Germany), unless stated otherwise. Plasmid DNA purification and gel extraction kits as well as restriction enzymes were purchased from NEB (Frankfurt am Main, Germany). Sanger sequencing was carried out by Eurofins (Ebersberg, Germany).

### Strains and plasmids

All strains and plasmids used in this study are listed in Table 1 and Table 2. The genome of the here used *V. natriegens* DSM759 Δ *dns* strain was sequenced on a NovaSeq 6000 platform with an S4 v1.5 4XP flow-cell (Illumina, San Diego, USA) in 200 cycles with a read length of 100 bp (paired-end). Identified mutations are provided in Table S6.

**Table 1:** *Escherichia coli* and *Vibrio natriegens* strains used in this study.

**Strain**	**Genotype**	**Reference/Origin**
NEB® 5-alpha	*E. coli fhuA2* Δ *(argF-lacZ)U169 phoA glnV44* Φ *80* Δ *(lacZ)M15 gyrA96 recA1 relA1 endA1 thi-1 hsdR17*	NEB ${}^{\mathrm{TM}}$
BL21	*E. coli fhuA2 [lon] ompT gal [dcm]* Δ *hsdS*	NEB ${}^{\mathrm{TM}}$
BL21 (DE3)	*E. coli fhuA2 [lon] ompT gal (* λ *DE3) [dcm]* Δ *hsdS*	NEB ${}^{\mathrm{TM}}$
DH5 α - λ *pir*	*E. coli* φ 80d*lac*Z Δ M15 Δ *(lacZYA-argF)U196 recA1 hsdR17 deoR thi-1 supE44 gyrA96 relA1/pir*	77
WM3064	*E. coli thrB1004 pro thi rpsL hsdS lacZ* Δ *M15 RP4-1360* Δ *(araBAD)567* Δ *dapA1341::[erm pir]*	77
*V. natriegens*	*V. natriegens* DSM759 (ATCC14048) Δ *dns*	73
*V. natriegens* Δ *lysC1*	*V. natriegens* DSM759 Δ *dns* Δ *lysC1*	This study
*V. natriegens* Δ *lysC2*	*V. natriegens* DSM759 Δ *dns* Δ *lysC2*	This study
*V. natriegens* Δ *lysC3*	*V. natriegens* DSM759 Δ *dns* Δ *lysC3*	This study
*V. natriegens* Δ *lysC1* Δ *lysC2*	*V. natriegens* DSM759 Δ *dns* Δ *lysC1* Δ *lysC2*	This study
*V. natriegens* Δ *lysC1* Δ *lysC2* Δ *lysC3*	*V. natriegens* DSM759 Δ *dns* Δ *lysC1* Δ *lysC2* Δ *lysC3*	This study
*V. natriegens* Δ *thrA*	*V. natriegens* DSM759 Δ *dns* Δ *thrA*	This study
*V. natriegens* Δ *metL*	*V. natriegens* DSM759 Δ *dns* Δ *metL*	This study
*V. natriegens* Δ *thrA* Δ *metL*	*V. natriegens* DSM759 Δ *dns* Δ *thrA* Δ *metL*	This study
*V. natriegens* Δ *lysC1* Δ *thrA* Δ *metL*	*V. natriegens* DSM759 Δ *dns* Δ *lysC1* Δ *thrA* Δ *metL*	This study
*V. natriegens* Δ *lysC2* Δ *thrA* Δ *metL*	*V. natriegens* DSM759 Δ *dns* Δ *lysC2* Δ *thrA* Δ *metL*	This study
*V. natriegens* Δ *lysC3* Δ *thrA* Δ *metL*	*V. natriegens* DSM759 Δ *dns* Δ *lysC3* Δ *thrA* Δ *metL*	This study
*V. natriegens* Δ *lysC1* Δ *lysC3* Δ *thrA* Δ *metL*	*V. natriegens* DSM759 Δ *dns* Δ *lysC1* Δ *lysC3* Δ *thrA* Δ *metL*	This study
*V. natriegens* Δ *asd1*	*V. natriegens* DSM759 Δ *dns* Δ *asd1*	This study
*V. natriegens* Δ *asd2*	*V. natriegens* DSM759 Δ *dns* Δ *asd2*	This study

**Table 2:** Plasmids used in this study.

**Plasmid**	**Relevant characteristics**	**Reference/Origin**
*In vitro studies*		
pET28a(+)	f1 origin, Kan ${}^{R}$ , T7 promoter	Novagen™
pET28-*Ec.lysC*	pET28a(+) derivative with N-terminal His-tagged *Ec.lysC*	This study
pET28-*Vn.lysC1*	pET28a(+) derivative with N-terminal His-tagged *Vn.lysC1*	This study
pET28-*Vn.lysC2*	pET28a(+) derivative with N-terminal His-tagged *Vn.lysC2*	This study
pET28-*Vn.lysC3*	pET28a(+) derivative with N-terminal His-tagged *Vn.lysC3*	This study
pCA24N-*Ec.thrA*	pCA24N derivative with N-terminal His-tagged *Ec.thrA*	78
pET28-*Vn.thrA*	pET28a(+) derivative with N-terminal His-tagged *Vn.thrA*	This study
pCA24N-*Ec.metL*	pCA24N derivative with N-terminal His-tagged *Ec.metL*	78
pET28-*Vn.metL*	pET28a(+) derivative with N-terminal His-tagged *Vn.metL*	This study
pCA24N-*Ec.asd*	pCA24N derivative with N-terminal His-tagged *Ec.asd*	78
pET28-*Vn.asd1*	pET28a(+) derivative with N-terminal His-tagged *Vn.asd1*	This study
pET28-*Vn.asd2*	pET28a(+) derivative with N-terminal His-tagged *Vn.asd2*	This study
pCA24N-*Ec.dapA*	pCA24N derivative with N-terminal His-tagged *Ec.dapA*	78
pET28-*Vn.dapA1*	pET28a(+) derivative with N-terminal His-tagged *Vn.dapA1*	This study
pET28-*Vn.dapA2*	pET28a(+) derivative with N-terminal His-tagged *Vn.dapA2*	This study
pCA24N-*Ec.dapB*	pCA24N derivative with N-terminal His-tagged *Ec.dapB*	78
*Strain engineering*		
pDM4	oriR6K, oriT, Cm ${}^{R}$ , *sacB*	28
pDM4- Δ Vn.lysC1	pDM4 derivative carrying 500 bp homologous sequences for the deletion of the *Vn.lysC1* gene	This study
pDM4- Δ Vn.lysC2	pDM4 derivative carrying 500 bp homologous sequences for the deletion of the *Vn.lysC2* gene	This study
pDM4- Δ Vn.lysC3	pDM4 derivative carrying 500 bp homologous sequences for the deletion of the *Vn.lysC3* gene	This study
pDM4- Δ Vn.thrA	pDM4 derivative carrying 500 bp homologous sequences for the deletion of the *Vn.thrA* gene	This study
pDM4- Δ Vn.metL	pDM4 derivative carrying 500 bp homologous sequences for the deletion of the *Vn.metL* gene	This study
pDM4- Δ Vn.asd1	pDM4 derivative carrying 500 bp homologous sequences for the deletion of the *Vn.asd1* gene	This study
pDM4- Δ Vn.asd2	pDM4 derivative carrying 500 bp homologous sequences for the deletion of the *Vn.asd2* gene	This study
pST_116	oriColE1, Cm ${}^{R}$ , Tet ${}^{R}$ , P ${}_{\mathrm{tet}}$ Cas9, P ${}_{\mathrm{J23106}}$ AcrIIA4, P ${}_{\mathrm{tac}}$ *Vc.tfox*	42
pST_116_ Δ Vn.lysC2	pST_116 derivative carrying gRNA targeting the *Vn.lysC2* locus	This study

**Table 3:** **Characterization of target enzymes putatively implicated in the L-lysine biosynthesis pathway in *V. natriegens*.** a

**Enzymatic activity**	**Enzyme name**	**Substrate**	**v** ${}_{\max}$ **[U mg** ${}^{\mathrm{-1}}$ **]**	**K** ${}_{M}$ **[mM]**	**K** ${}_{I}$ **[mM]**	**Inhibited by**^f^
AK	Ec.LysC	L-aspartate^b^	7.16 ± 1.64	1.92 ± 0.07	-	Lys (2 ± 1 %)
	Vn.LysC1		14.43 ± 2.81	5.35 ± 0.40	-	Lys (5 ± 0 %)
	Vn.LysC2		7.64 ± 1.67	5.52 ± 0.61	-	-
	Vn.LysC3		n.d.	n.d.	n.d.	n.m.

AK-HD	Ec.ThrA	L-aspartate^b^	1.51 ± 0.67	3.94 ± 0.33	-	Thr (14 ± 5 %)
		ASA^c^	5.54 ± 0.25	0.14 ± 0.02	-	n.m.
	Vn.ThrA	L-aspartate^b^	0.59 ± 0.03	17.99 ± 1.36	-	Thr (80 ± 3 %)
		ASA^c^	5.79 ± 1.33	0.35 ± 0.00	-	n.m.
	Ec.MetL	L-aspartate^b^	4.00 ± 0.49	4.53 ± 1.10	-	-
		ASA^c^	18.90 ± 1.77	0.19 ± 0.02	0.85 ± 0.06	n.m.
	Vn.MetL	L-aspartate^b^	3.12 ± 0.61	12.79 ± 1.83	-	-
		ASA^c^	30.61 ± 0.59	0.89 ± 0.16	0.20 ± 0.04	n.m.

ASD^d^	Ec.Asd	L-aspartyl phosphate	15.63 ± 1.69	n.m.	n.m.	n.m.
	Vn.Asd1		21.74 ± 1.37	n.m.	n.m.	n.m.
	Vn.Asd2		2.43 ± 0.24	n.m.	n.m.	n.m.

DHDPS	Ec.DapA	ASA^c^	25.11 ± 3.97	0.27 ± 0.02	-	Lys (13 ± 2 %)
	Vn.DapA1		19.18 ± 4.20	0.20 ± 0.01	-	Lys (5 ± 1 %)
	Vn.DapA2		0.07 ± 0.01^e^	n.sat.	n.sat.	Lys (83 ± 9 %)

a Comparison of kinetic parameters and regulation of *V. natriegens* DSM759 and *E. coli* MG1655 enzymes. Experiments were carried out at pH 7.5 and 37 ${}^{\circ }$ C. Experimental data was fitted to the Michaelis-Menten or substrate inhibition model to determine kinetic parameters. n.d. – no activity detectable, n.m. – not measured, n.sat. – not saturated, n ≥ 2.

b Kinetic parameters of AK activity were determined on 0.012-50 mM L-aspartate as substrate.

c Kinetic parameters of HD and DHDPS activities were determined on 0.001-1 mM L-aspartate semialdehyde (ASA) as substrate.

d Due to *in situ* production of the substrate L-aspartyl phosphate, kinetic parameters of ASD enzymes could not be measured (n.m.). As v ${}_{\max}$ , the specific ASD activity on 50 mM substrate is given.

e No substrate saturation was observed (n.sat.). The specific DHDPS activity in presence of the highest tested substrate concentration of 1 mM ASA is given as v ${}_{\max}$ .

f AFAA causing inhibition of enzymatic activity are listed with the corresponding residual specific activity in parentheses (relative to the control condition without amino acid supplementation). Substrates: 50 mM L-aspartate or 0.5 mM ASA; inhibitory AFAA: 5 mM; Lys – L-lysine; Thr – L-threonine.

### Media

LB media (LB Broth (Luria/Miller), Carl Roth) and LB agar plates (LB Agar (Luria/Miller), Carl Roth) were used for cloning procedures, protein production and cell recovery from glycerol stocks (25% v/v) stored at −80 ${}^{\circ }$ C. For liquid LB-cultures of *V. natriegens* strains, the NaCl concentration was increased to 15 g L ${}^{-1}$ (LBN media). Where required, antibiotics were added in the following final concentrations: 50 mg L ${}^{-1}$ kanamycin sulphate and 35 mg L ${}^{-1}$ chloramphenicol for *E. coli*; 3 mg L ${}^{-1}$ chloramphenicol for *V. natriegens*.

For RNA sequencing experiments, cells were cultivated in VN mineral medium 23 containing 10 g L ${}^{-1}$ glucose, 5 g L ${}^{-1}$ (NH ${}_{4}$ ) ${}_{2}$ SO ${}_{4}$ , 15 g L ${}^{-1}$ NaCl, 1 g L ${}^{-1}$ KH ${}_{2}$ PO ${}_{4}$ , 1 g L ${}^{-1}$ K ${}_{2}$ HPO ${}_{4}$ , 0.25 g L ${}^{-1}$ MgSO ${}_{4}$ , 10 mg L ${}^{-1}$ CaCl ${}_{2}$ , 16.4 mg L ${}^{-1}$ FeSO ${}_{4}\cdot$ 7 H ${}_{2}$ O and trace elements (10 mg L ${}^{-1}$ MnSO ${}_{4}\cdot$ H ${}_{2}$ O, 0.3 mg L ${}^{-1}$ CuSO ${}_{4}\cdot$ 5 H ${}_{2}$ O, 1 mg L ${}^{-1}$ ZnSO ${}_{4}\cdot$ 7 H ${}_{2}$ O and 0.02 mg L ${}^{-1}$ NiCl ${}_{2}\cdot$ 6 H ${}_{2}$ O). VN mineral media agar plates with 4 g L ${}^{-1}$ glucose were prepared by addition of 16 g L ${}^{-1}$ agar-agar.

### Construction of *V. natriegens* DSM759 deletion strains

Marker-less chromosomal deletions of the *Vn.lysC1, Vn.lysC2, Vn.lysC3, Vn.thrA, Vn.metL, Vn.asd1* and *Vn.asd2* genes were performed *via* homologous recombination using pDM4-derived suicide vectors allowing for chloramphenicol selection and *sacB*-based counter selection 28, 79. pDM4 vectors carrying 500 bp sequences homologous to the up- and downstream regions for each target gene are listed in Table 2. For backbone linearization, 2 μ g of the pDM4 vector were digested with the restriction enzymes XbaI and SacI (NEB). Homologous regions were amplified by PCR from *V. natriegens* DSM759 chromosomal DNA using Q5 polymerase (NEB) according to manufacturer’s protocol and the primers listed in Table S1. The fragments were purified by gel extraction (Monarch® Spin DNA Gel Extraction Kit, NEB). Both homologous DNA fragments were simultaneously inserted into the digested backbone by isothermal assembly using the NEBuilder® HiFi DNA Assembly Master Mix (NEB) according to manufacturer’s protocol. Constructed plasmids were transformed into chemically competent *E. coli* DH5 α - λ *pir* and verified by Sanger sequencing.

For chromosomal deletions, the pDM4 plasmids were transformed into chemically competent cells of the DAP-auxotrophic donor strain *E. coli* WM3064 and conjugated into *V. natriegens* DSM759. Transconjugants were streaked onto LB agar plates containing 3 μ g mL ${}^{-1}$ chloramphenicol (LB+Chm plates) for selection of isolates with chromosomal integration of the suicide vector and counter selection of the donor strain. To verify successful plasmid integration, isolates were replica-plated onto LB+Chm as well as LB+Chm plates additionally containing 25% sucrose. Single colonies sensitive to sucrose were selected and incubated in LBN media (no additives) at 30 ${}^{\circ }$ C, 220 rpm. After 4–5 h, 3 μ L of the liquid culture was streaked onto LB agar plates containing 25% sucrose and incubated at room temperature for 24–48 h. Colonies were replica plated on LB and LB+Chm plates. Colonies sensitive to chloramphenicol were selected and successful chromosomal modification was verified *via* diagnostic PCR (primers, see Table S1) using DreamTaq polymerase (ThermoFisher Scientific) according to the manufacturer’s protocol. Multiple gene deletions were introduced sequentially. For the construction of strains which were anticipated to be auxotrophic for the DAP, the host strains were first transformed with the pSB64.1 plasmid 80, and kanamycin (250 μ g mL ${}^{-1}$ ) and DAP (0.25 g L ${}^{-1}$ ) were added for selection of crossover events. All strains were eventually cured from the helper plasmid.

Diverging from the pDM4-based deletion strategy, the *lysC2* gene in strain *V. natriegens* Δ *lysC2* Δ *thrA* Δ *metL* was deleted using the NT-CRISPR method following the protocol of Stukenberg *et al.*, 2021 42. The guide RNA (gRNA) sequence was designed using the Cas-Designer tool (Bae *et al.*, 2014 81; Park *et al.*, 2015 82; available at http://www.rgenome.net/cas-designer/) and selected based on an out-of-frame score greater than 66. The template DNA (tDNA) fragment used for homologous recombination was generated by overlap-extension PCR, according to the corresponding protocol.io procedure 83. Plasmids and primers used are listed in Table 2 and Table S1. The subsequent steps were carried out as described in the NT-CRISPR protocol. Correct deletion of the target gene was confirmed by diagnostic PCR (primers listed in Table S1) using DreamTaq DNA polymerase (Thermo Fisher Scientific) according to the manufacturer’s instructions.

### Agar plate screening

To phenotype the *V. natriegens* DSM759 Δ *dns* reference strain and the constructed deletion mutants, the strains were precultured in LBN medium at 30 ${}^{\circ }$ C and 220 rpm (Ecotron, Infors) for about 4 h until an optical density at 600 nm (OD ${}_{\mathrm{600 nm}}$ ) of 1–2 was reached. For putative diaminopimelic acid (DAP)-auxotrophic strains, the LBN medium was additionally supplemented with 0.25 g L ${}^{-1}$ DAP. Cells were harvested by centrifugation (4500 g, 10 min, RT) and washed twice with 9 g L ${}^{-1}$ NaCl. The final OD ${}_{\mathrm{600 nm}}$ was adjusted to 0.01 and 4 μ L of the cell suspensions were spotted onto VN mineral media agar plates supplemented with 0.05 g L ${}^{-1}$ L-lysine, 0.2 g L ${}^{-1}$ L-threonine, 0.2 g L ${}^{-1}$ L-methionine and/or 0.25 g L ${}^{-1}$ DAP in different combinations. Plates were incubated at 30 ${}^{\circ }$ C and colony formation was analysed after 24 h.

### Construction of plasmids for protein production and protein purification procedures

pET28 and pCA24N 78 vectors harbouring genes encoding the target enzymes were used for production of N-terminally 6x-His-tagged proteins (Table 2). pET28 plasmids harbouring *Ec.lysC, Vn.lysC1, Vn.lysC2, Vn.lysC3, Vn.thrA* and *Vn.asd1* genes were constructed *via* restriction-ligation-based cloning. The target genes were amplified by PCR from chromosomal DNA of *E. coli* MG1655 or *V. natriegens* DSM759, respectively, using Q5 polymerase (NEB) according to manufacturer’s protocol. The primers introduced NdeI and EcoRI restriction sites into the 5’ and 3’ overhangs of the amplified DNA fragments (Table S1). The pET28 backbone vector and the inserts were digested with the restriction enzymes NdeI and EcoRI (NEB). After DNA purification by gel extraction (Monarch® Spin DNA Gel Extraction Kit, NEB), the backbone and inserts were ligated using T4 DNA ligase (NEB) according to the manufacturer’s protocol. pET28 plasmids harbouring the *Vn.metL, Vn.asd2, Vn.dapA1* and *Vn.dapA2* genes were constructed *via* isothermal assembly. The target genes were amplified by PCR from *V. natriegens* DSM759 chromosomal DNA using Q5 polymerase (NEB) according to the manufacturer’s protocol and the primers listed in Table S1. For backbone linearization, 2 μ g of the pET28 vector were digested with the restriction enzymes NdeI and BamHI (NEB). The fragments were purified by gel extraction (Monarch® Spin DNA Gel Extraction Kit, NEB). For isothermal assembly of the backbone and inserts, the NEBuilder® HiFi DNA Assembly Master Mix (NEB) was used according to manufacturer’s protocol. All constructed pET28 vectors were transformed into chemically competent NEB® 5-alpha (NEB) and verified by Sanger sequencing.

For gene expression, pET28 and pCA24N plasmids were transformed into chemically competent BL21 (DE3) and BL21 cells, respectively. A volume of 50 mL LB medium with 50 μ g/mL kanamycin was inoculated at an initial OD ${}_{\mathrm{600 nm}}$ of 0.2 with an overnight LB-preculture. The cultures were incubated at 37 ${}^{\circ }$ C and 220 rpm in 250 mL shake flasks. Heterologous gene expression was induced with 1 mM isopropyl- β -D-thiogalactopyranosid (IPTG) at an OD ${}_{\mathrm{600 nm}}$ of 0.6. After incubation at 25 ${}^{\circ }$ C and 220 rpm for 20 h, cells were harvested by centrifugation (4500 g, 10 min, 4 ${}^{\circ }$ C) and the cell pellets were stored at −20 ${}^{\circ }$ C until further analysis.

For protein purification, cell pellets were thawed on ice, resuspended in 1.5 mL HEPES buffer (50 mM HEPES, 300 mM NaCl, pH 7.5) and disrupted using an ultrasonic disintegrator (4 times 30 s, 20% amplitude, UDS 751, TOPAS, Germany). Cell debris was removed from the soluble fraction by centrifugation (13000 g, 15 min, 4 ${}^{\circ }$ C). His-tagged proteins were purified using the TALON® Superflow™ resin. For binding, the crude cell extract was added to the pre-washed resin and incubated at room temperature for 20 min in a tube rotator (VWR). Two washing steps, firstly with HEPES buffer (50 mM HEPES, 300 mM NaCl, pH 7.5) and secondly with HEPES buffer additionally containing 15 mM imidazole were carried out. The resin-bound protein was eluted with 500 μ L of HEPES buffer (50 mM HEPES, 300 mM NaCl, pH 7.5) containing 250 mM imidazole. The concentration of the purified protein was determined *via* the Bradford assay (ROTI®Quant, Carl Roth).

### Enzymatic assays

Aspartate kinase (AK), homoserine dehydrogenase (HD), aspartate semialdehyde dehydrogenase (Asd) and dihydrodipicolinate synthase (DHDPS) activities were determined in the biosynthetic sense of the reactions *via* enzymatic assays with purified enzyme. Specific activities were determined by measuring the characteristic absorption of NAD(P)H at 340 nm, which was either directly converted by the target enzyme or *via* coupled enzymatic assays connecting the target enzymatic activity to NAD(P)H oxidation using auxiliary enzymes. The assays were conducted in 96-well flat-bottomed microtiter plates with a final reaction volume of 250 μ L. The reaction kinetics were monitored using a microplate reader (NanoQuant Plate™, Infinite® 200 PRO, TECAN) at 37 ${}^{\circ }$ C. One unit (U) is defined as the amount of enzyme that catalyses the conversion of 1 μ mol NAD(P)H per minute at pH 7.5. For the determination of kinetic parameters, the experimental data was fitted to the Michaelis-Menten or substrate inhibition model by non-linear regression (curve fitting tool, MATLAB R2021a). K ${}_{M}$ is defined as the apparent Michaelis-Menten constant [mM] and v ${}_{\max}$ is the apparent maximum specific activity [U mg ${}^{-1}$ ].

Aspartate kinase activities of Ec.LysC, Vn.LysC1, Vn.LysC2, Vn.LysC3, Ec.ThrA, Vn.ThrA, Ec.MetL and Vn.MetL were assayed by coupling the ATP production of the AK reaction to NADH oxidation via pyruvate kinase and lactate dehydrogenase 84, 85. The reaction mixture contained 50 mM HEPES, 5 mM MgCl ${}_{2}$ , 50 mM KCl, 1 mM phosphoenolpyruvate, 0.25 mM NADH, 2 mM ATP, 2 U mL ${}^{-1}$ pyruvate kinase, 2 U mL ${}^{-1}$ lactate dehydrogenase and appropriate amounts of the purified enzyme. For the determination of kinetic parameters, the reactions were started by the addition of L-aspartate in variable concentrations (0.012–50 mM). To elucidate allosteric inhibition by L-lysine, L-threonine, L-isoleucine, L-methionine or L-ectoine, 5 mM of each amino acid was added to the reaction mix and the reactions were started with 50 mM L-aspartate.

Assays to determine homoserine dehydrogenase activities of Ec.ThrA, Vn.ThrA, Ec.MetL and Vn.MetL were carried out by direct measurement of NADPH oxidation. The reaction mix contained 50 mM HEPES, 5 mM MgCl ${}_{2}$ , 50 mM KCl, 0.25 mM NADPH and appropriate amounts of purified enzyme. The reactions were started by the addition of L-aspartate- β -semialdehyde in variable concentrations (0.001–1 mM).

Aspartate semialdehyde dehydrogenase activities of Ec.Asd, Vn.Asd1 and Vn.Asd2 were assayed by following the oxidation of NADPH during the reduction of aspartyl phosphate 85. As the substrate L-aspartyl phosphate is unstable and not commercially available, it was produced *in situ* by action of purified Ec.LysC. The reaction mix contained 50 mM HEPES, 5 mM MgCl ${}_{2}$ , 50 mM KCl, 3 mM ATP, 50 mM L-aspartate, 0.1 mg mL ${}^{-1}$ purified Ec.LysC and appropriate amounts of purified Asd. The reactions were started by the addition of 0.25 mM of the cofactor NADPH.

Assays to determine dihydrodipicolinate synthase activities of Ec.DapA, Vn.DapA1 and Vn.DapA2 were conducted by coupling the dihydrodipicolinate formation of the DHDPS to NADH oxidation by dihydrodipicolinate reductase (DHDPR) 86, 87. The reaction mix contained 100 mM HEPES, 40 mM pyruvate, 0.25 mM NADH, 20 μ g mL ${}^{-1}$ purified DHDPR (Ec.DapB) and appropriate amounts of purified DHDPS. The reactions were started by addition of the substrate L-aspartate- β -semialdehyde. For the determination of kinetic parameters, L-aspartate- β -semialdehyde concentrations were varied between 0.001 and 1 mM. To elucidate allosteric inhibition, 0.5 mM of the substrate were used and 5 mM of the putative inhibitor L-lysine were added.

### RNA sequencing and transcriptome analysis

For a first preculture, 5 mL of LBN medium was inoculated with a single colony of *V. natriegens* DSM759 Δ *dns* and incubated overnight at 30 ${}^{\circ }$ C, 220 rpm. For adaption to mineral media, a second preculture was cultivated in 25 mL VN medium (200 mM MOPS buffer). These cultures were inoculated at an initial OD ${}_{\mathrm{600 nm}}$ of 0.2 and incubated until an OD ${}_{\mathrm{600 nm}}$ of 0.5 was reached (37 ${}^{\circ }$ C, 220 rpm). Cells were harvested *via* centrifugation (4500 g, 10 min, RT), washed with 9 g L ${}^{-1}$ NaCl and used for inoculation of the main culture at an initial OD ${}_{\mathrm{600 nm}}$ of 0.1. Main cultures were incubated in 10 mL VN medium (0 mM MOPS, 37 ${}^{\circ }$ C, 220 rpm) supplemented with either 20 mM L-lysine, L-threonine, L-isoleucine, L-methionine or L-ectoine. The reference condition did not contain any supplements. When the main cultures reached an OD ${}_{\mathrm{600 nm}}$ of 0.5, 3 mL of the cell suspensions were mixed with 6 mL of RNAprotect® Bacteria Reagent (QIAGEN, Germany) by vortexing and incubated for 5–15 min at RT to stabilize RNA transcripts. Subsequently, stabilized cultures were centrifuged for 10 min at 5000 g. Most of the supernatant was decanted, the pellet resuspended in the remaining liquid and centrifuged again for 2 min at 5000 g. For RNA isolation, the Quick-RNA Fungal/Bacterial Miniprep Kit (Zymo Research, Freiburg, Germany) including DNase I treatment was used according to the manufacturer’s protocol. RNA was eluted with 50 μ L DNase/RNase-free water, aliquoted and stored at −80 ${}^{\circ }$ C until further use. The experiment was carried out in triplicates.

RNA integrity and total RNA concentration were measured with a FragmentAnalyzer (Advanced Analytics/Agilent, USA) using the Agilent DNF-471 RNA Kit (15 nt).

Total RNA aliquots were thawed, diluted to equal concentrations and handed to DRESDEN-concept Genome Center (Dresden, Germany) for mRNA enrichment by rRNA depletion (NEBNext® rRNA Depletion Kit (Bacteria), NEB), library preparation (NEBNext® Ultra™ II Directional RNA Library Prep Kit for Illumina®, NEB) and paired-end RNA sequencing on a NovaSeq 6000 platform with an S4 v1.5 4XP flow-cell (Illumina, San Diego, USA) in 200 cycles with a read length of 100 bp.

For transcriptome analysis, first a genome index for the *V. natriegens* RefSeq assembly GCF_001456255.1 was built using Rsubread 2.18.0 88. Reads were aligned to the genome index employing Rsubread with the default arguments. The count matrix was obtained using the gtf annotation file (NCBI RefSeq GCF_001456255.1-RS_2024_04_26) and featureCounts of Rsubread (deviating parameters: paired-end, reversely stranded, no-multi mapping reads counted, feature types counted: gene, transcript, CDS, exon). Only genes with at least 10 counts in more than three samples were considered for subsequent differential gene expression analysis using DESeq2 89 with the default arguments. The design formula used for DESeq2 accounted for different treatments and replicates. For plotting, shrunken fold changes (shrinkage estimator: apeglm 90) and adjusted p values (Benjamini-Hochberg, 91) were used. Gene Set Enrichment Analysis was performed using clusterProfiler 92. GO terms were gathered from the gtf annotation file from NCBI and bundled into an organism database using AnnotationForge 93. KEGG pathway assignments were obtained from the KEGG database 36. The minimum gene set size was set to 10, the maximum to 500 with an overall significance level of 0.05. To reduce redundancy, similar GO terms were grouped using the Wang algorithm in the simplify function of clusterProfiler (employing the GOSemSim package 94) with a similarity cutoff of 0.8 and, if necessary, further grouped manually.

## SUPPLEMENTAL MATERIAL

All supplemental data for this article are available online at http://microbialcell.com/researcharticles/2026a-straube-microbial-cell/. 

## CONFLICT OF INTEREST

None declared.

## ABBREVIATIONS

AFAA – L-aspartate family amino acids

AK – aspartate kinase

ASA – L-aspartate semialdehyde

ASD – aspartate semialdehyde dehydrogenase

DAP – diaminopimelic acid

DHDPS – dihydrodipicolinate synthase

GO – gene ontology

LFC – log2 fold change

SAM – S-adenosyl methionine

UTR – untranslated region

HD – homoserine dehydrogenase
