# Supplementary material for: Genetic make-up and regulation of the L-lysine biosynthesis pathway in Vibrio natriegens
Source: Microb Cell. 2026 Feb 3;13:44–62. doi: 10.15698/mic2026.02.867 (PMC12925634; doi:10.15698/mic2026.02.867)
Supplement: Supplementary file 1 [file mic-13-044-s01.pdf]

Supplemental Material

Genetic make-up and regulation of the L-lysine biosynthesis pathway in *Vibrio natriegens*

Elly Straube[1], Johannes Radde[1], Thi Van Anh Tran[1], Negin Keihani Yazdi[1], Rubén Crespo Blanco[1], Ha Thanh Le[2], Cláudio J. R. Frazão[1], Thomas Walther[1*]

Author affiliations:

[1] Institute of Natural Materials Technology, TU Dresden, 01062 Dresden, Germany

[2] School of Chemistry and Life Science, Hanoi University of Science and Technology, 1 Dai Co Viet, Hanoi, Vietnam

*** Correspondence:**
Thomas Walther
thomas_walther@tu-dresden.de

Table S1: Primers used in this study.

Primer	Sequence (5'→3')
Construction of pDM4 vectors	
Vn.lysC1-up-fw	GCATGCAAGATCTATCTAGACTCCTTGTTTGACGTTACTTAAC
Vn.lysC1-up-rv	TATTGTGAGATGAACAGCGTTGAGTTCTCCCACGAC
Vn.lysC1-down-fw	AGAAGTCGTGGGAGAACTCAACGCTGTTCATCTCACAATATAA
Vn.lysC1-down-rv	CTCGAGTACGCGTCACTAGTGCGATGATTATAATGCCATTATG
Vn.lysC2-up-fw	GCATGCAAGATCTATCTAGATGCGGCTCTAGAAGG
Vn.lysC2-up-rv	AAACTACCTTGTTAGCGAAACAGTCTTCACCTTCCC
Vn.lysC2-down-fw	TCTTGGGAAGGTGAAGACTGTTTCGCTAACAAGGTAG
Vn.lysC2-down-rv	CTCGAGTACGCGTCACTAGTAAATATGTCAAACACTTTCTTTG
Vn.lysC3-up-fw	GCATGCAAGATCTATCTAGACATCTTAATAAGGAAAATTAATAATGATC
Vn.lysC3-up-rv	CGTCTTGTTGTGTAATTGACGTTAAGCCTTTTTTGATTGG
Vn.lysC3-down-fw	CCAATCAAAAAAGGCTTAACGTCAATTACACAACAAGACGTGA
Vn.lysC3-down-rv	CTCGAGTACGCGTCACTAGTTGCTGGTTCCGTTTCTG
Vn.thrA-up-fw	TACCCGCATGCAAGATCTATCATCAACTGCACGCCACC
Vn.thrA-up-rv	GGAGGAAGGGCAGTAATGAGTTCAAGTGATATGGATG
Vn.thrA-down-fw	CTCATTACTGCCCTTCCTCCAGTGTATC
Vn.thrA-down-rv	TATCAAGCTTATCGATACCGGGAGTGTGATCACACTTAAATTG
Vn.metL-up-fw	TACCCGCATGCAAGATCTATGGTGGGGCAACTGTATTG
Vn.metL-up-rv	TTTTGAGTGTTATTAACTCCTATTGCTGTGC
Vn.metL-down-fw	GGAGTTAATAACACTCAAAACCCCGGAATTC
Vn.metL-down-rv	GCGTCACTAGTGGGGCCCTTCTGATTTGATTTCTTTGATGATGGAATG
Vn.asd1-up-fw	GCATGCAAGATCTATCTAGAAAGAAGGAATAAACCTAACGAG
Vn.asd1-up-rv	TCATCAAAAATAGAGAAACGCGCCTTAACTCTCCATGTATTA
Vn.asd1-down-fw	ATACATGGAGAGTTAAGGCGCGTTTCTCTATTTTTGATG
Vn.asd1-down-rv	CTCGAGTACGCGTCACTAGTGCTATCTTCAAGATTCTAAT
Vn.asd2-up-fw	TGTGGAATCCCGGGAGAGCTCTTGTTGACAACAGCGCATTAAAG
Vn.asd2-up-rv	CTCTCAGAGCTCATTGACTTACCTCAATCTGAAAAC
Vn.asd2-down-fw	AAGTCAATGAGCTCTGAGAGTTATTGAAAC
Vn.asd2-down-rv	GCGTCACTAGTGGGGCCCTTCTAGAGTTGATTCGATAGATAGCAAG
NT-CRISPR	
gRNA-Vn.lysC2-fw	GTCCACGCTCACTTACCGGAGCAC
gRNA-Vn.lysC2-rv	AAACGTGCTCCGGTAAGTGAGCGT
OE-PCR_Vn.lysC2-up-fw	ACAGGTGCGTTTTATGCAATATC
OE-PCR_Vn.lysC2-up-rv	TTCGTAAACTACCTTGTTAGCGAAACAGTCTTCACCTTCCCAAG
OE-PCR_Vn.lysC2-down-fw	CTTGGGAAGGTGAAGACTGTTTCGCTAACAAGGTAGTTTACG
OE-PCR_Vn.lysC2-down-rv	TATCCTAAAAGGTTCTGACCAC
Construction of pET28 vectors	
Ec.lysC-fw	CCACCACATATGATGTCTGAAATTGTTGTCTCCAAATTTGGC
Ec.lysC-rv	CCACCAGAATTCTTACTCAAACAAATTACTATGCAGTTTTTGCACC
Vn.lysC1-fw	CCACCACATATGGTGAGCGCATTTAACGTAGC
Vn.lysC1-rv	CCACCAGAATTCTTATTTTTCAAATAGCTCAGCATGCAG
Vn.lysC2-fw	CCACCACATATGGTGAAAAAGCCCCTTATCGTGCA
Vn.lysC2-rv	CCACCAGAATTCTCACCCTATTAGGGCATGTTTTTGC
Vn.lysC3-fw	CCACCACATATGATGACTTTTACCGTAGAAAAAATCGGCG

Vn.lysC3-rv	CCACCAGAATTCTTACGCAACGTCTTCTATCGACTCAG
Vn.thrA-fw	CCACCACATATGATGCGAGTATTGAAGTTTGGCGG
Vn.thrA-rv	CCACCAGAATTCTTATACCCCTAGTTTCCAACCTAAAGTACG
Vn.metL-fw	CTGGTGCCGCGCGGCAGCCATATGACTGTACAACGTCAG
Vn.metL-rv	GTCGACGGAGCTCGAATTCGGATCCTTAGAATAGCCCAGCAAG
Vn.asd1-fw	CCACCACATATGATGAGAGTTGGTTTAGTTGGTTG
Vn.asd1-rv	CCACCAGAATTCTTATGCTTTCTCTTGAAGGATAA
Vn.asd2-fw	CTGGTGCCGCGCGGCAGCCATATGATGAGCCAACAATATAATGTTG
Vn.asd2-rv	AAGCTTGTCGACGGAGCTCGAATTCTTAGTAGTAATCGCGAATAAG
Vn.dapA1-fw	CTGGTGCCGCGCGGCAGCCATATGATGTTTTCAGGAAGTATCGTAGC
Vn.dapA1-rv	TGTCGACGGAGCTCGAATTCTCAATCTTTATAAATACGGGCGT
Vn.dapA2-fw	CTGGTGCCGCGCGGCAGCCATATGATGAAGCTCGACGGTATTTTTG
Vn.dapA2-rv	AAGCTTGTCGACGGAGCTCGAATTCCTATCGTAACGCTGCATC
Deletion strain verification primers	
ver-ΔVn.lysC1-fw	TACGGTGAGGGGGAATAAAG
ver-ΔVn.lysC1-rv	GGTGGTATTGGTGAGCG
ver-ΔVn.lysC2-fw	GGGTAAAGCGGTAGAAGTTAACG
ver-ΔVn.lysC2-fw (NT-CRISPR)	GGTTTGAACTGGCAAGAATG
ver-ΔVn.lysC2-rv	AACCACATACTCCCTTAGCAGG
ver-ΔVn.lysC3-fw	GAATCAGAGCTCGATCAAGG
ver-ΔVn.lysC3-rv	GCGAACGCTTATCTTTGTCG
ver-ΔVn.thrA-fw	GCTAATGATACCAAGCTCTTC
ver-ΔVn.thrA-rv	CAACGCTTCCCAATATTAAAGC
ver-ΔVn.metL-fw	CGCTAAAACGGAAGAGCATG
ver-ΔVn.metL-rv	GATACAAGTCAGGTGCGG
ver-ΔVn.asd1-fw	GGAAGATCGAGTTTAGCAGTGAAC
ver-ΔVn.asd1-rv	GCGTGTGTTGCGTATTTTCC
ver-ΔVn.asd2-fw	AACGGTCTACGTGCAGATC
ver-ΔVn.asd2-rv	GAAGACGCAGTCTGCTTC
Plasmid verification primers	
ver-pDM4-fw	CACTTAACGGCTGACATGG
ver-pDM4-rv	GCTCCAGTGGCTTCTGTTTC
ver-pET28-fw	ATGCGTCCGGCGTAGA
ver-pET28-rv	CTAGTTATTGCTCAGCGGT

Table S2: Gene symbols used in this study.

Enzyme	Gene name	Locus tag	Previous locus tag	Chr
Aspartate kinase (AK)	*lysC1*	PN96_RS00270	PN96_00270	1
Aspartate kinase (AK)	*lysC2*	PN96_RS01175	PN96_01145	1
Aspartate kinase (AK)	*lysC3*	PN96_RS05310	PN96_05230	1
Aspartate kinase-homoserine dehydrogenase (AK-HD)	*thrA*	PN96_RS11205	PN96_11055	1
Aspartate kinase-homoserine dehydrogenase (AK-HD)	*metL*	PN96_RS14980	PN96_14770	1
Aspartate-semialdehyde dehydrogenase	*asd1*	PN96_RS03220	PN96_03170	1
Aspartate-semialdehyde dehydrogenase	*asd2*	PN96_RS02955	PN96_02900	1
Dihydrodipicolinate synthase	*dapA1*	PN96_RS02525	PN96_02485	1
Dihydrodipicolinate synthase	*dapA2*	PN96_RS19840	PN96_19580	2
Dihydrodipicolinate reductase	*dapB*	PN96_RS11310	PN96_11160	1
Tetrahydrodipicolinate succinylase	*dapD*	PN96_RS02000	PN96_01975	1
N-succinyl-diaminopimelate aminotransferase	*argD*	PN96_RS14820	PN96_14620	1
N-succinyl-L-diaminopimelate desuccinylase	*dapE*	PN96_RS02545	PN96_02505	1
Diaminopimelate epimerase	*dapF*	PN96_RS14075	PN96_13885	1
Diaminopimelate decarboxylase	*lysA*	PN96_RS14070	PN96_13880	1
Homoserine kinase	*thrB*	PN96_RS11200	PN96_11050	1
L-threonine synthase	*thrC*	PN96_RS11195	PN96_11045	1
L-threonine dehydratase	*ilvA*	PN96_RS13635	PN96_13455	1
Acetohydroxyacid synthase	*ilvI*	PN96_RS11720	PN96_11565	1
Acetohydroxyacid synthase	*ilvH*	PN96_RS11715	PN96_11560	1
Acetohydroxyacid synthase	*ilvM*	PN96_RS13645	PN96_13465	1
Acetohydroxyacid synthase	*RS13650*	PN96_RS13650	PN96_13470	1
Acetohydroxyacid isomeroreductase	*ilvC*	PN96_RS13345	PN96_13170	1
Dihydroxyacid dehydratase	*ilvD*	PN96_RS13640	PN96_13460	1
Branched-chain amino acid aminotransferase	*RS21300*	PN96_RS21300	PN96_21010	2
Homoserine O-succinyltransferase	*metA*	PN96_RS05150	PN96_05065	1
O-succinylhomoserine-lyase	*metB*	PN96_RS14975	PN96_14765	1
Cystathionine-β-lyase	*metC1*	PN96_RS07710	PN96_07605	1
Cystathionine-β-lyase	*metC2*	PN96_RS22415	PN96_22110	2
Homocysteine transmethylase	*metE1*	PN96_RS03895	PN96_03850	1
Homocysteine transmethylase	*metE2*	PN96_RS19260	PN96_19000	2
Homocysteine transmethylase	*metH*	PN96_RS00265	PN96_00265	1
L-methionine adenosyltransferase	*metK*	PN96_RS00900	PN96_00880	1
Diaminobutyrate pyruvate aminotransferase	*ectB1*	PN96_RS05300	PN96_05220	1
Diaminobutyrate pyruvate aminotransferase	*ectB2*	PN96_RS04065	PN96_04015	1
Diaminobutyrate acetyltransferase	*ectA*	PN96_RS05295	PN96_05215	1
Ectoine synthase	*ectC1*	PN96_RS05305	PN96_05225	1
Ectoine synthase	*ectC2*	PN96_RS16045	PN96_15825	2

Based on information obtained from BioCyc [35] and KEGG databases [36]. In case of duplicate genes encoding putative isozymes, a consecutive numbering of the gene symbols was introduced.

A

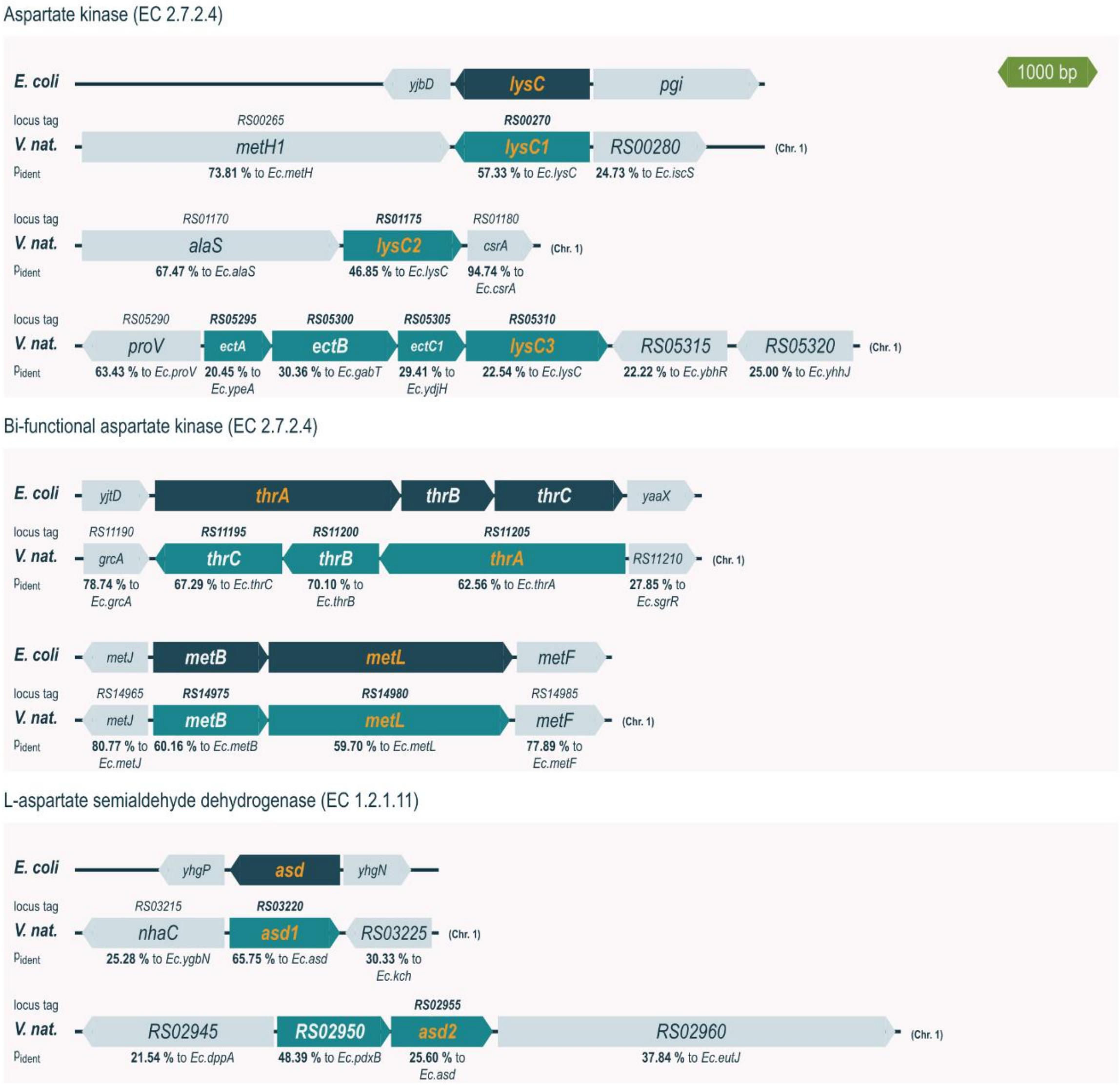

Aspartate kinase (EC 2.7.2.4)
1000 bp
E. coli
yjbD
lysC
pgi
locus tag
RS00265
RS00270
V. nat.
metH1
lysC1
RS00280
(Chr. 1)
pident
73.81 % to Ec.metH
57.33 % to Ec.lysC
24.73 % to Ec.iscS
locus tag
RS01170
RS01175
RS01180
V. nat.
alaS
lysC2
csrA
(Chr. 1)
pident
67.47 % to Ec.alaS
46.85 % to Ec.lysC
94.74 % to Ec.csrA
locus tag
RS05290
RS05295
RS05300
RS05305
RS05310
V. nat.
proV
ectA
ectB
ectC1
lysC3
RS05315
RS05320
(Chr. 1)
pident
63.43 % to Ec.proV
20.45 % to Ec.ypeA
30.36 % to Ec.gabT
29.41 % to Ec.ydjH
22.54 % to Ec.lysC
22.22 % to Ec.ybhR
25.00 % to Ec.yhhJ
Bi-functional aspartate kinase (EC 2.7.2.4)
E. coli
yjtD
thrA
thrB
thrC
yaaX
locus tag
RS11190
RS11195
RS11200
RS11205
V. nat.
grcA
thrC
thrB
thrA
RS11210
(Chr. 1)
pident
78.74 % to Ec.grcA
67.29 % to Ec.thrC
70.10 % to Ec.thrB
62.56 % to Ec.thrA
27.85 % to Ec.sgrR
E. coli
metJ
metB
metL
metF
locus tag
RS14965
RS14975
RS14980
RS14985
V. nat.
metJ
metB
metL
metF
(Chr. 1)
pident
80.77 % to Ec.metJ
60.16 % to Ec.metB
59.70 % to Ec.metL
77.89 % to Ec.metF
L-aspartate semialdehyde dehydrogenase (EC 1.2.1.11)
E. coli
yhgP
asd
yhgN
locus tag
RS03215
RS03220
V. nat.
nhaC
asd1
RS03225
(Chr. 1)
pident
25.28 % to Ec.ygbN
65.75 % to Ec.asd
30.33 % to Ec.kch
locus tag
RS02955
V. nat.
RS02945
RS02950
asd2
RS02960
(Chr. 1)
pident
21.54 % to Ec.dppA
48.39 % to Ec.pdxB
25.60 % to Ec.asd
37.84 % to Ec.eutJ

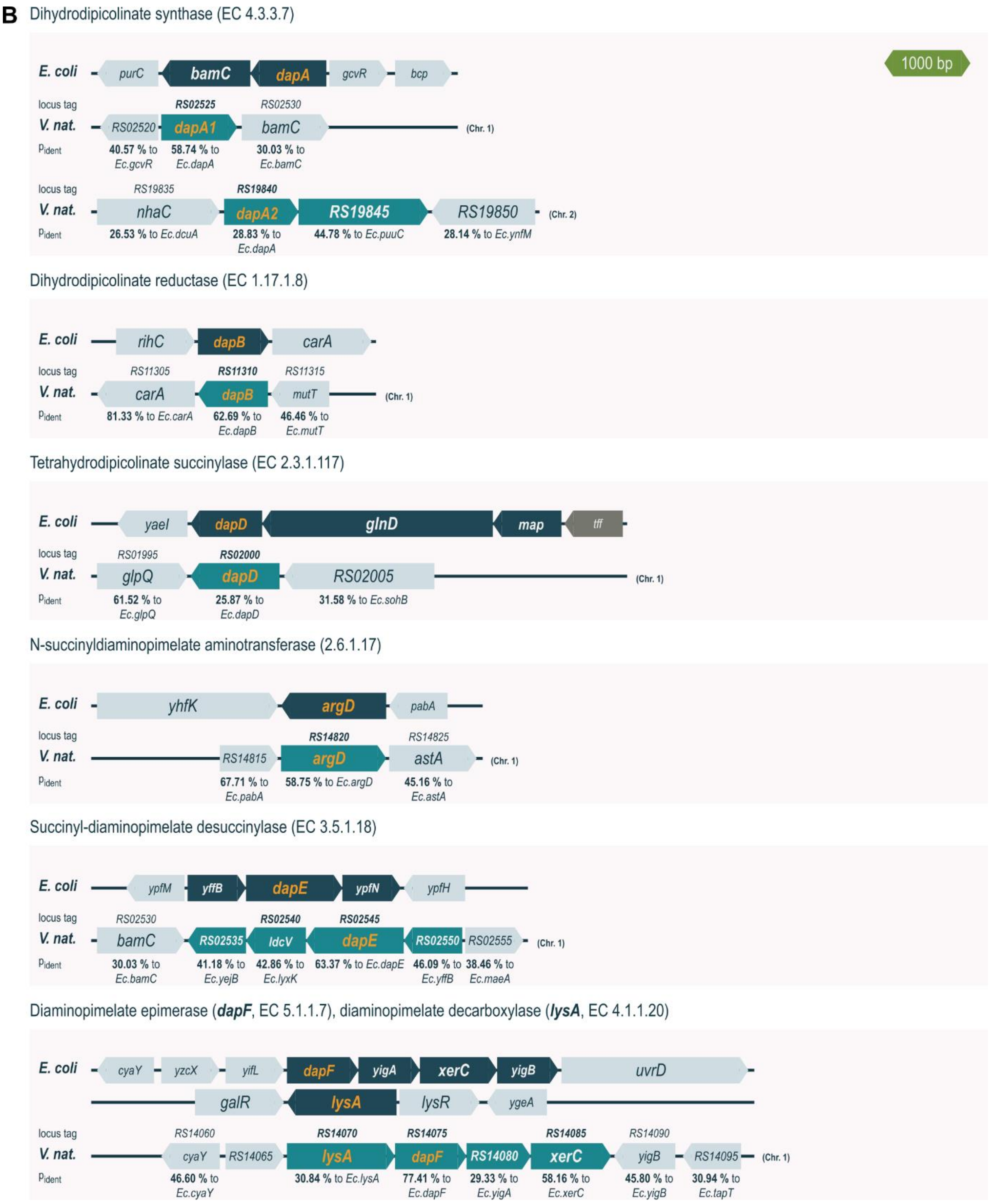
B
Dihydrodipicolinate synthase (EC 4.3.3.7)
1000 bp
E. coli
purC
bamC
dapA
gcvR
bcp
locus tag
RS02525
RS02530
V. nat.
RS02520
dapA1
bamC
(Chr. 1)
pident
40.57 % to Ec.gcvR
58.74 % to Ec.dapA
30.03 % to Ec.bamC
locus tag
RS19835
RS19840
V. nat.
nhaC
dapA2
RS19845
RS19850
(Chr. 2)
pident
26.53 % to Ec.dcuA
28.83 % to Ec.dapA
44.78 % to Ec.puuC
28.14 % to Ec.ynfM
Dihydrodipicolinate reductase (EC 1.17.1.8)
E. coli
rihC
dapB
carA
locus tag
RS11305
RS11310
RS11315
V. nat.
carA
dapB
mutT
(Chr. 1)
pident
81.33 % to Ec.carA
62.69 % to Ec.dapB
46.46 % to Ec.mutT
Tetrahydrodipicolinate succinylase (EC 2.3.1.117)
E. coli
yaeI
dapD
glnD
map
tff
locus tag
RS01995
RS02000
V. nat.
glpQ
dapD
RS02005
(Chr. 1)
pident
61.52 % to Ec.glpQ
25.87 % to Ec.dapD
31.58 % to Ec.sohB
N-succinyldiaminopimelate aminotransferase (2.6.1.17)
E. coli
yhfK
argD
pabA
locus tag
RS14820
RS14825
V. nat.
RS14815
argD
astA
(Chr. 1)
pident
67.71 % to Ec.pabA
58.75 % to Ec.argD
45.16 % to Ec.astA
Succinyl-diaminopimelate desuccinylase (EC 3.5.1.18)
E. coli
ypfM
yffB
dapE
ypfN
ypfH
locus tag
RS02530
RS02540
RS02545
V. nat.
bamC
RS02535
ldcV
dapE
RS02550
RS02555
(Chr. 1)
pident
30.03 % to Ec.bamC
41.18 % to Ec.yejB
42.86 % to Ec.lyxK
63.37 % to Ec.dapE
46.09 % to Ec.yffB
38.46 % to Ec.maeA
Diaminopimelate epimerase (dapF, EC 5.1.1.7), diaminopimelate decarboxylase (lysA, EC 4.1.1.20)
E. coli
cyaY
yzcX
yifL
dapF
yigA
xerC
yigB
uvrD
galR
lysA
lysR
ygeA
locus tag
RS14060
RS14070
RS14075
RS14085
RS14090
V. nat.
cyaY
RS14065
lysA
dapF
RS14080
xerC
yigB
RS14095
(Chr. 1)
pident
46.60 % to Ec.cyaY
30.84 % to Ec.lysA
77.41 % to Ec.dapF
29.33 % to Ec.yigA
58.16 % to Ec.xerC
45.80 % to Ec.yigB
30.94 % to Ec.tapT

C Homoserine kinase (***thrB***, EC 2.7.1.39), L-threonine synthase (***thrC***, EC 4.2.3.1)

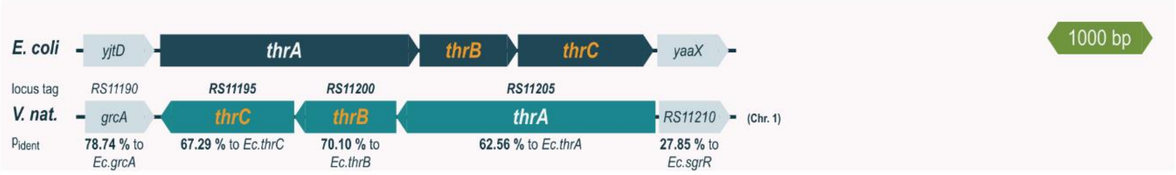

D L-threonine dehydratase (***ilvA***, EC 4.3.1.19), Acetohydroxy acid synthase (***ilvM***, EC 2.2.1.6), Dihydroxy acid dehydratase (***ilvD***, EC 4.2.1.9)

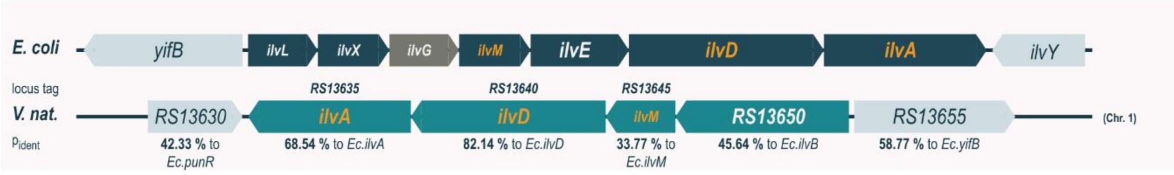

Acetohydroxy acid synthase (EC 2.2.1.6)

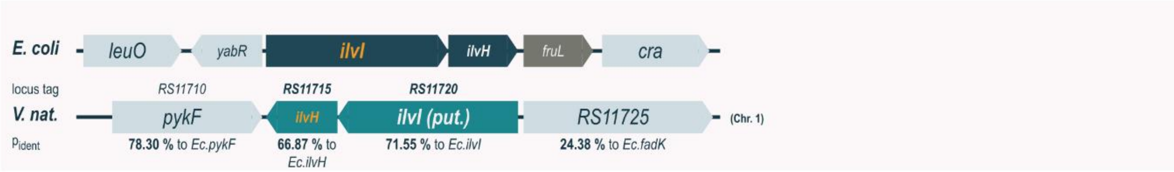

Acetohydroxy acid isomeroreductase (EC 1.1.1.86)

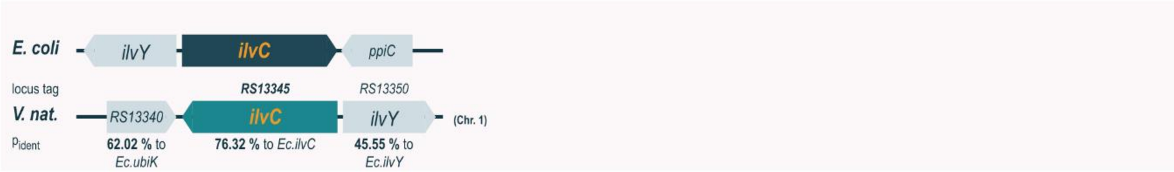

Branched chain amino acid aminotransferase (EC 2.6.1.42)

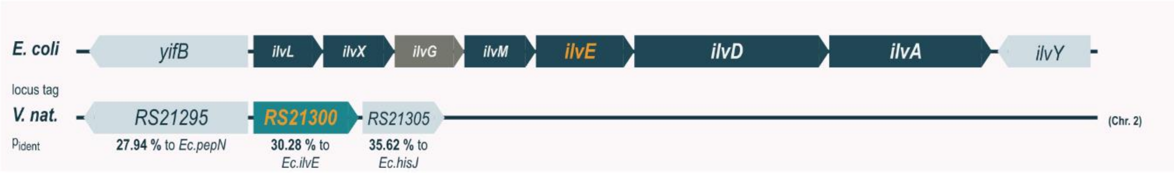

E Diaminobutyrate pyruvate aminotransferase (***ectB***, EC 2.6.1.76), L-2,4-diaminobutyric acid acetyltransferase (***ectA***, EC 2.3.1.178), Ectoine synthase (***ectC***, EC 4.2.1.108)

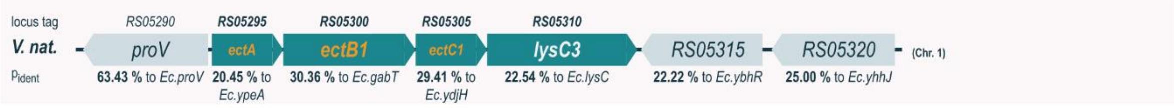

Diaminobutyrate pyruvate aminotransferase (EC 2.6.1.76)

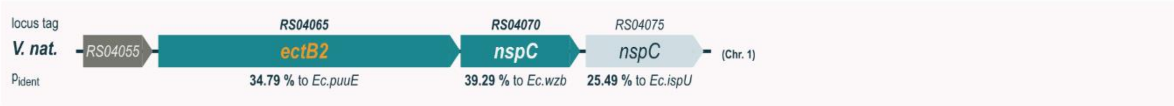

Ectoine synthase (EC 4.2.1.108)

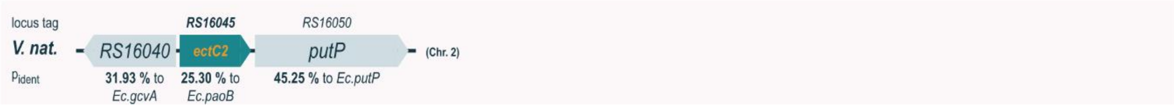

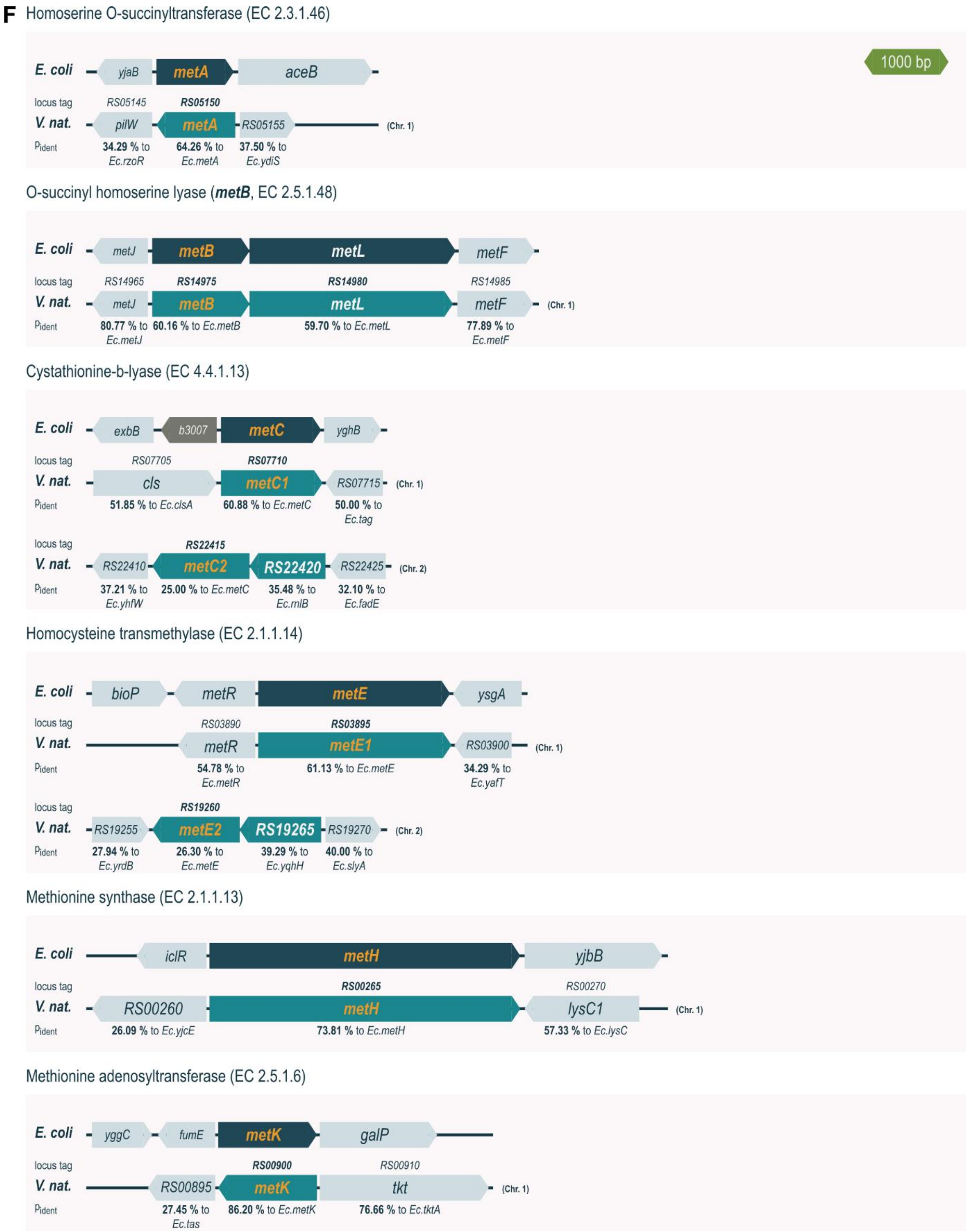
F
Homoserine O-succinyltransferase (EC 2.3.1.46)
1000 bp
E. coli
yjaB
metA
aceB
locus tag
RS05145
RS05150
V. nat.
pilW
metA
RS05155
(Chr. 1)
Pident
34.29 % to Ec.rzoR
64.26 % to Ec.metA
37.50 % to Ec.ydiS
O-succinyl homoserine lyase (metB, EC 2.5.1.48)
E. coli
metJ
metB
metL
metF
locus tag
RS14965
RS14975
RS14980
RS14985
V. nat.
metJ
metB
metL
metF
(Chr. 1)
Pident
80.77 % to Ec.metJ
60.16 % to Ec.metB
59.70 % to Ec.metL
77.89 % to Ec.metF
Cystathionine-b-lyase (EC 4.4.1.13)
E. coli
exbB
b3007
metC
yghB
locus tag
RS07705
RS07710
V. nat.
cls
metC1
RS07715
(Chr. 1)
Pident
51.85 % to Ec.clsA
60.88 % to Ec.metC
50.00 % to Ec.tag
locus tag
RS22415
V. nat.
RS22410
metC2
RS22420
RS22425
(Chr. 2)
Pident
37.21 % to Ec.yhfW
25.00 % to Ec.metC
35.48 % to Ec.rnlB
32.10 % to Ec.fadE
Homocysteine transmethylase (EC 2.1.1.14)
E. coli
bioP
metR
metE
ysgA
locus tag
RS03890
RS03895
V. nat.
metR
metE1
RS03900
(Chr. 1)
Pident
54.78 % to Ec.metR
61.13 % to Ec.metE
34.29 % to Ec.yafT
locus tag
RS19260
V. nat.
RS19255
metE2
RS19265
RS19270
(Chr. 2)
Pident
27.94 % to Ec.yrdB
26.30 % to Ec.metE
39.29 % to Ec.yqhH
40.00 % to Ec.slyA
Methionine synthase (EC 2.1.1.13)
E. coli
iclR
metH
yjbB
locus tag
RS00265
RS00270
V. nat.
RS00260
metH
lysC1
(Chr. 1)
Pident
26.09 % to Ec.yjcE
73.81 % to Ec.metH
57.33 % to Ec.lysC
Methionine adenosyltransferase (EC 2.5.1.6)
E. coli
yggC
fumE
metK
galP
locus tag
RS00900
RS00910
V. nat.
RS00895
metK
tkt
(Chr. 1)
Pident
27.45 % to Ec.tas
86.20 % to Ec.metK
76.66 % to Ec.tktA

Figure S1: Comparison of the operon organisation of the L-lysine and related AFAAs biosynthesis pathways in *V. natriegens* and *E. coli*. Operon structure is shown **(A)** for genes of the shared entry of the AFAA biosynthesis pathway from L-aspartate to L-aspartate-semialdehyde/homoserine as well as **(B)** for genes included in the L-lysine biosynthesis pathway, **(C)** the L-threonine, **(D)** the L-isoleucine, **(E)** the L-ectoine and **(F)** the L-methionine pathway. Data obtained from BioCyc [35] and KEGG databases [36]. Genes are referred to by their gene symbol, if available, or by their locus tag in the format ”RS12345” based on the RefSeq reference genome (BioSample ID SAMN03178087). In case of duplicate genes encoding putative isozymes, a consecutive numbering of the gene symbols was introduced. Protein sequence alignment was performed using the NCBI BLAST® tool (blastp algorithm) comparing the amino acid sequence encoded by a given *V. natriegens* gene to the *E. coli* MG655 database (taxid: 511145). Percentage of calculated amino acid sequence identity if given below each *V. natriegens* DSM759 gene symbol (yellow text – genes pertaining to the associated enzymatic activity; medium and dark blue box – genes of same operon; grey box – neighbouring genes; scale bar: 1000 bp).

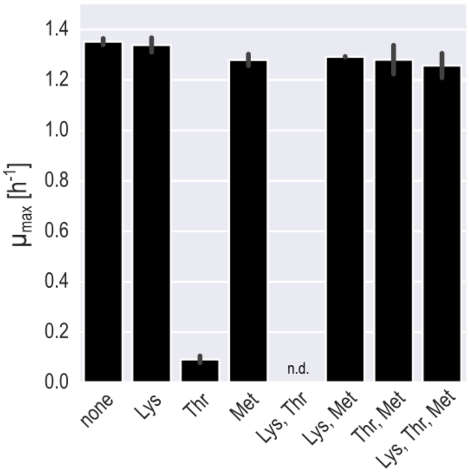

Figure S2: Growth behaviour of *V. natriegens* DSM759 *Δdns ΔmetL* in liquid culture in the presence of L-lysine (Lys), L-threonine (Thr) and/or L-methionine (Met). The maximum specific growth rate μ_{max} was calculated from the exponential growth phase observed during the first 10 hours of cultivation (25 mL VN mineral medium with 10 g L^{-1} glucose and 200 mM MOPS, 250 mL baffled shake flasks, 37 °C, 220 rpm, 20 mM of each Lys, Thr and/or Met, n.d. – no growth detectable, n=2).

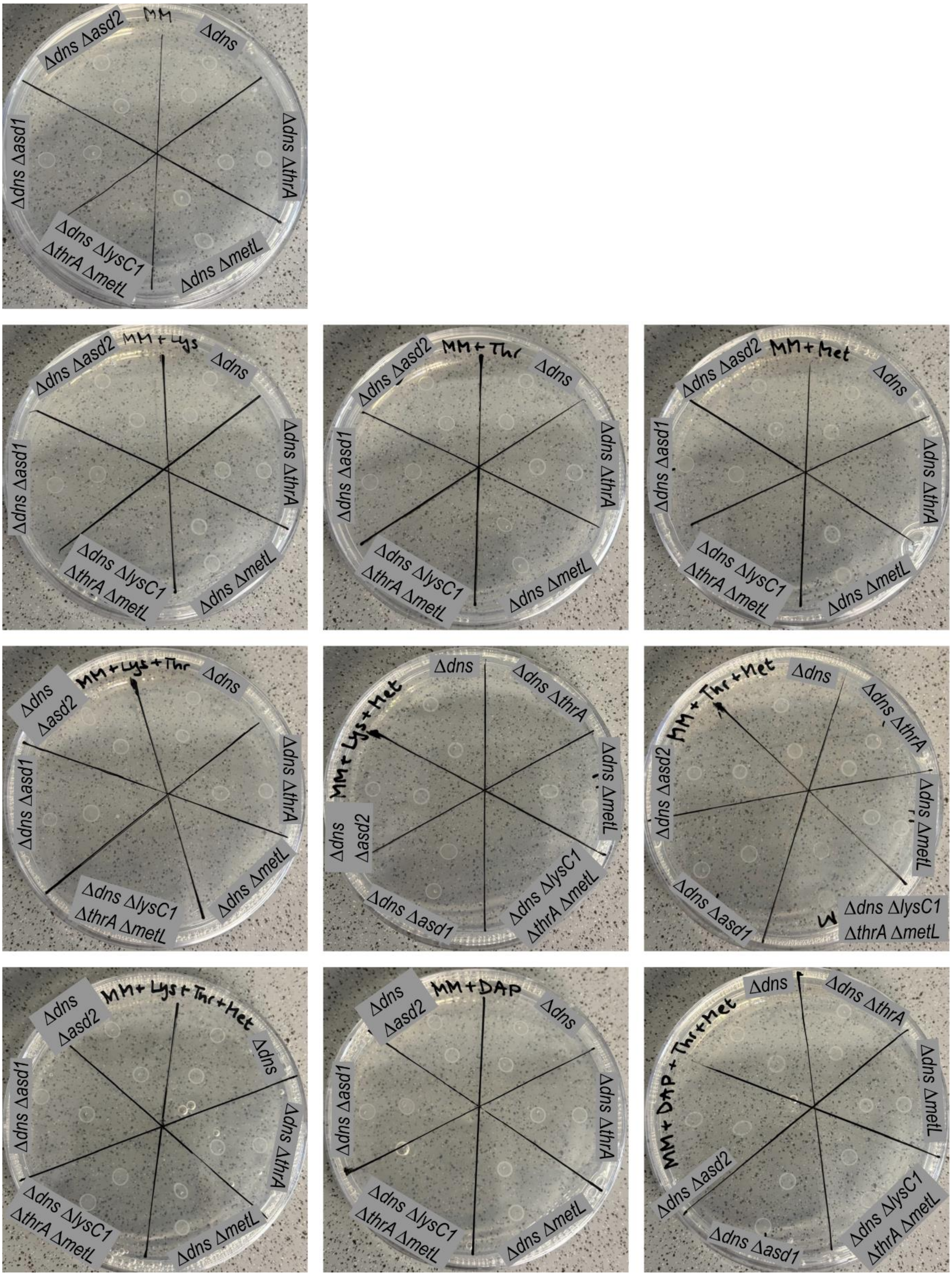

Figure S3: Exemplary VN mineral media agar plates used for phenotyping of L-lysine biosynthesis pathway gene deletion mutants. Two spots each (4 µL, OD = 0.01) of the *V. natriegens* DSM759 *Δdns* reference strain and the constructed deletion mutants were applied to the agar plates (supplemented with 0.05 g L^{-1} L-lysine (Lys), 0.2 g L^{-1} L-threonine (Thr), 0.2 g L^{-1} L-methionine (Met) and/or 0.25 g L^{-1} DAP). Plates were incubated at 30 °C and colony formation was analysed after 24 h.

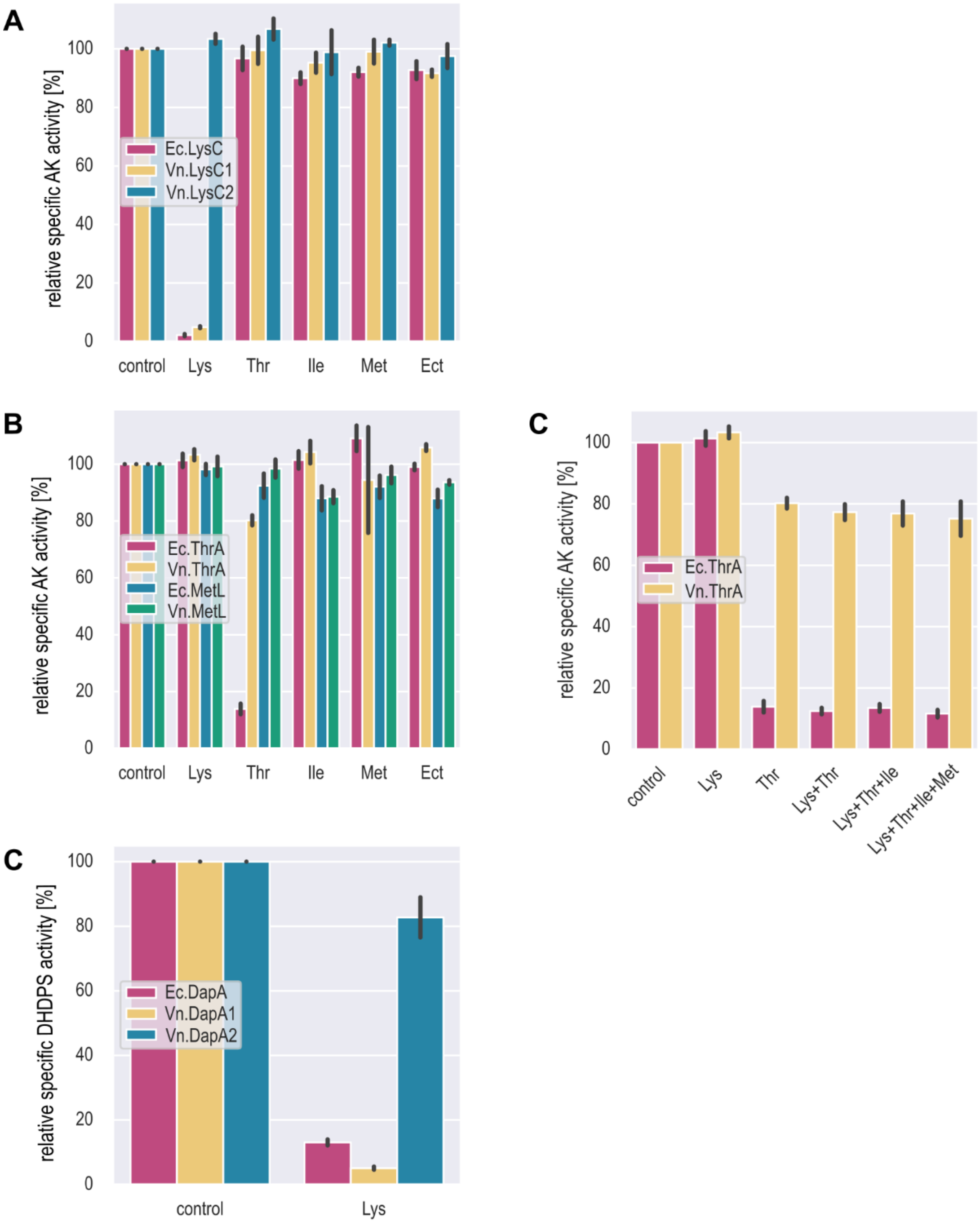

Figure S4: Inhibition profiles of *V. natriegens* and *E. coli* AK, AK-HD and DHDPS isozymes in response to AFAA addition. (A) The relative specific activities of mono-functional AK isozymes Ec.LysC, Vn.LysC1 and Vn.LysC2 as well as **(B)** of bi-functional AK-HD enzymes Ec.ThrA, Vn.ThrA, Ec.MetL and Vn.MetL are determined on 50 mM L-aspartate as substrate in the presence of L-lysine (Lys), L-threonine (Thr), L-Isoleucine (Ile), L-methionine (Met) or L-ectoine (Ect). **(C)** Inhibition profiles of Ec.ThrA and Vn.ThrA in response multiple AFAA supplementation. **(D)** The relative specific activities of DHDPS enzymes Ec.DapA, Vn.DapA1 and Vn.DapA2 on 0.5 mM ASA are shown in the presence of L-lysine (pH 7.5; 37 °C; 5 mM of each Lys, Thr, Ile, Met or Ect).

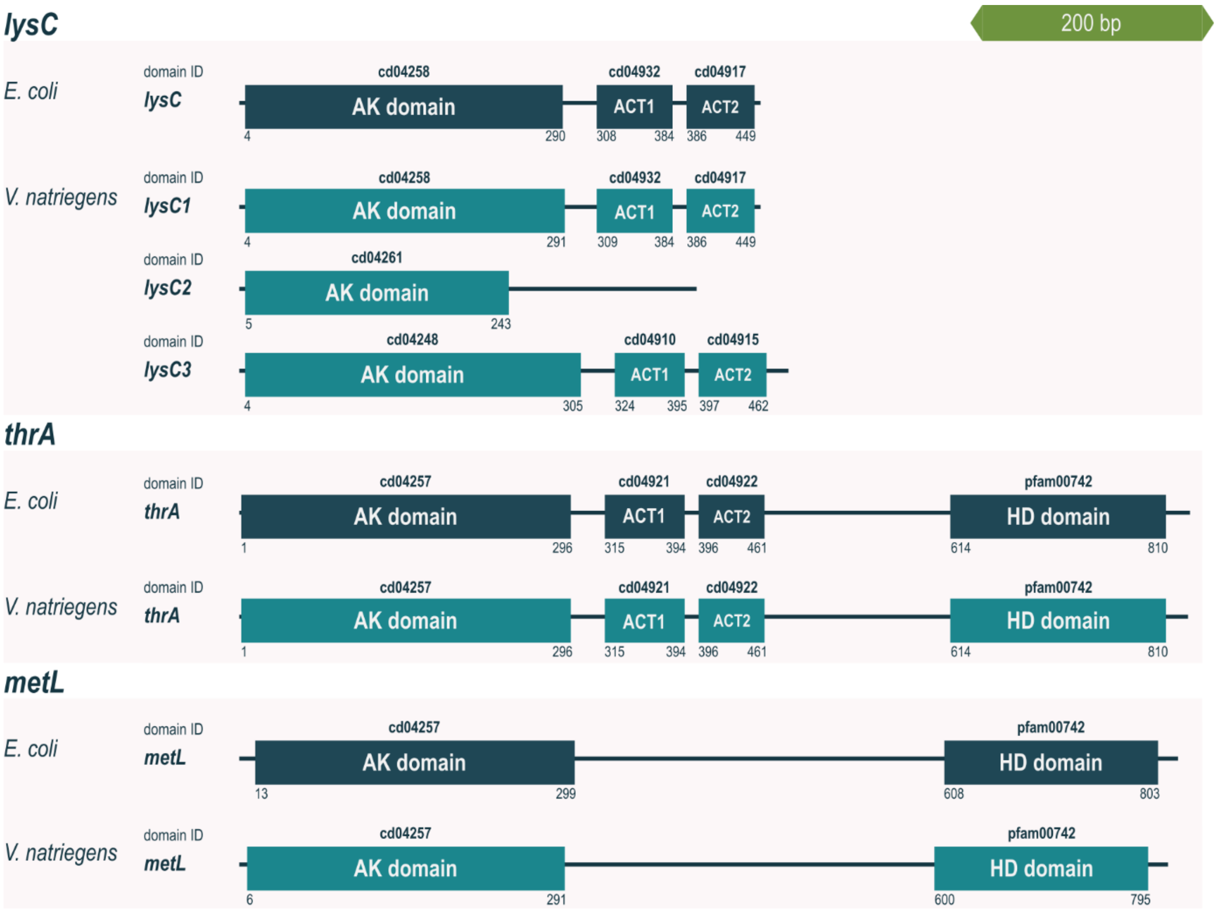

Figure S5: Predicted conserved domains of *V. natriegens* and *E. coli* AK and AK-HD enzymes. AA sequence data obtained from KEGG databases and conserved regions were analysed using the NCBI CD-search tool.

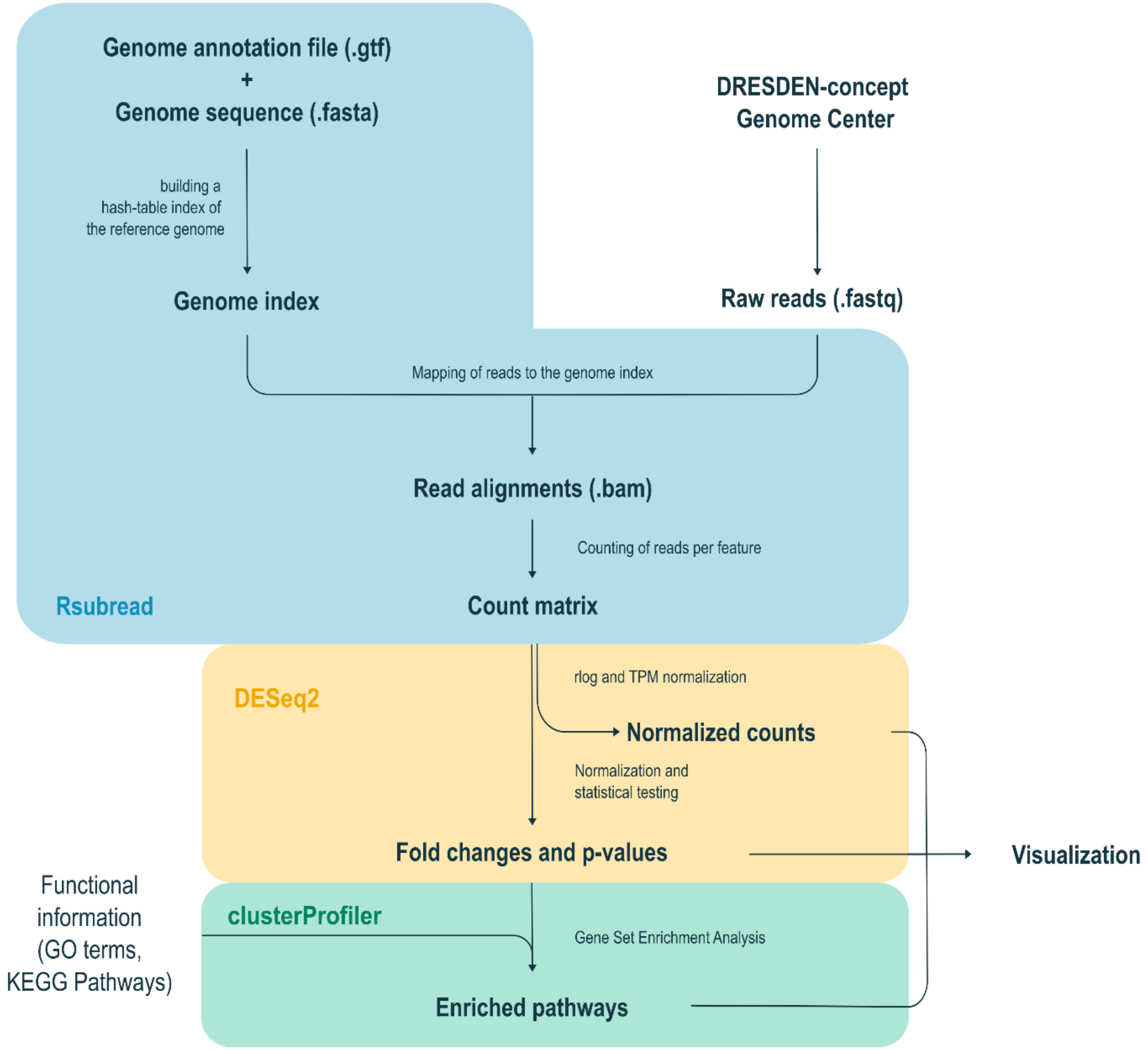

Figure S6: Transcriptome data analysis workflow applied in this study. Coloured boxes denote the R packages that were used for the respective steps. Blue: Rsubread [87]; yellow: DESeq2 [88]; green: cluster-Profiler [91]. R packages used for visualizations are not shown.

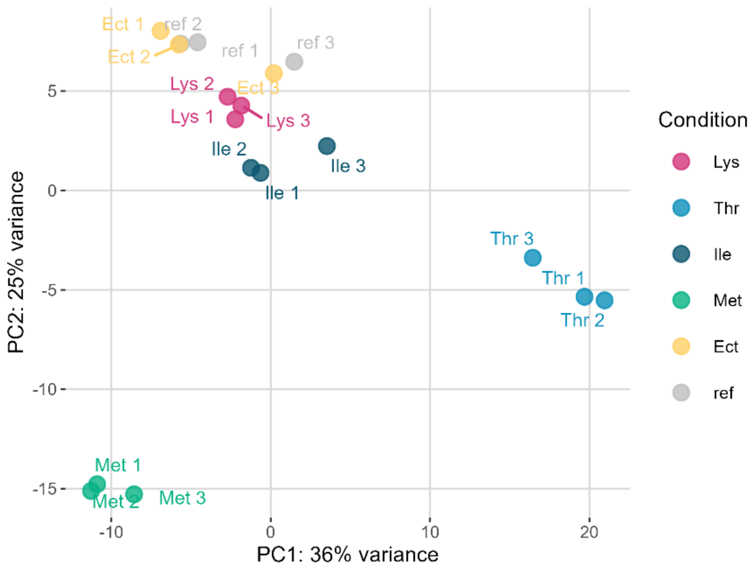

Figure S7: Assessment of inter- and intragroup variability of RNA-sequencing data. Principal component analysis (PCA) scatter plot of the three replicates for the reference and supplemented amino acid conditions (L-lysine (Lys), L-threonine (Thr), L-isoleucine (Ile), L-methionine (Met) and L-ectoine (Ect)). PCA was performed on rlog-transformed reads per gene counts using the plotPCA function of DESeq2 [88]. The percentages on the x- and y-axis represent the principal components, explaining 36 % and 25 % of the variance, respectively.

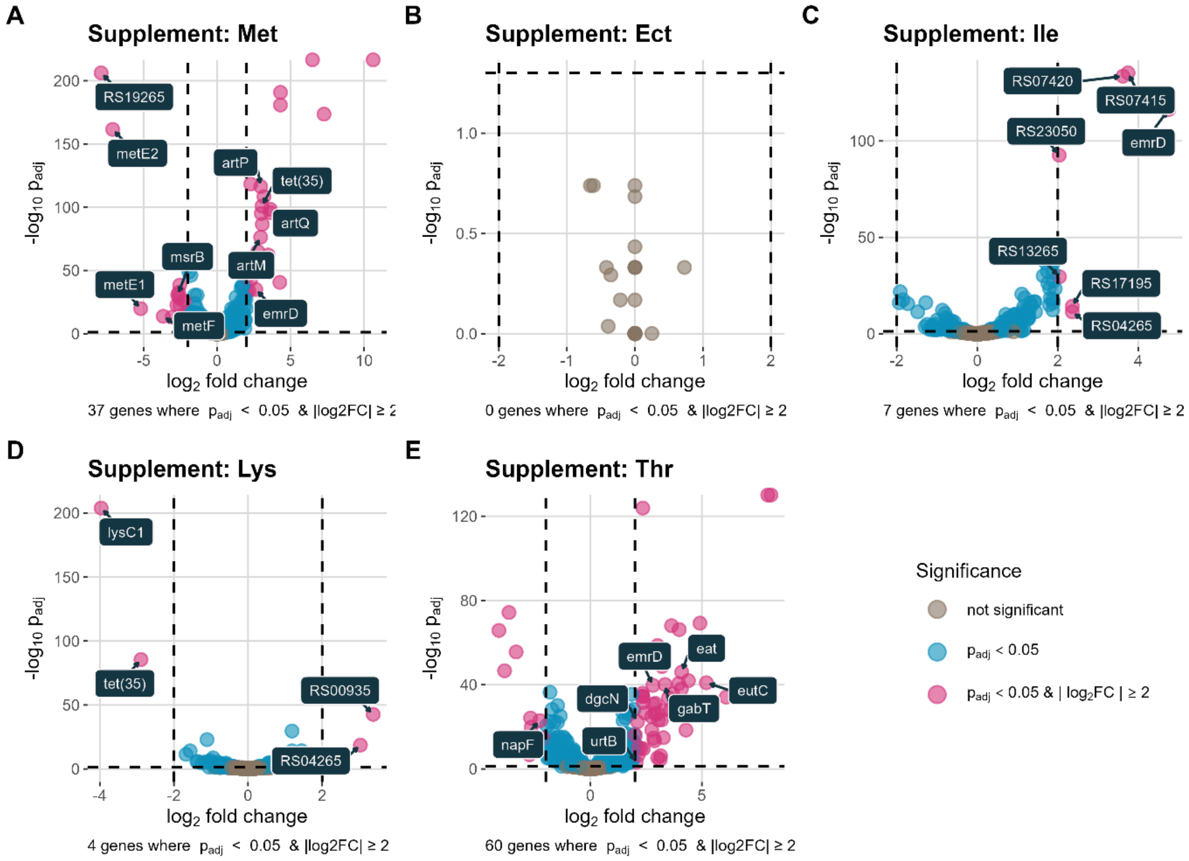

Figure S8: Identification of differentially expressed genes (DEGs) for each supplementation condition. Volcano plots depict the distribution of genes according to their change of expression (LFC, x-axis) and the respective statistical significance ($-\log_{10}p_{adj}$, y-axis) for each supplementation condition compared to the reference. DEGs, which meet the cut-off for p_{adj} < 0.05 (vertical dashed line), are coloured in blue. DEGs, which also meet the cut-off for |LCF| ≥ 2 (horizontal dashed lines), are depicted in red. Non-significantly expressed genes are shown in grey.

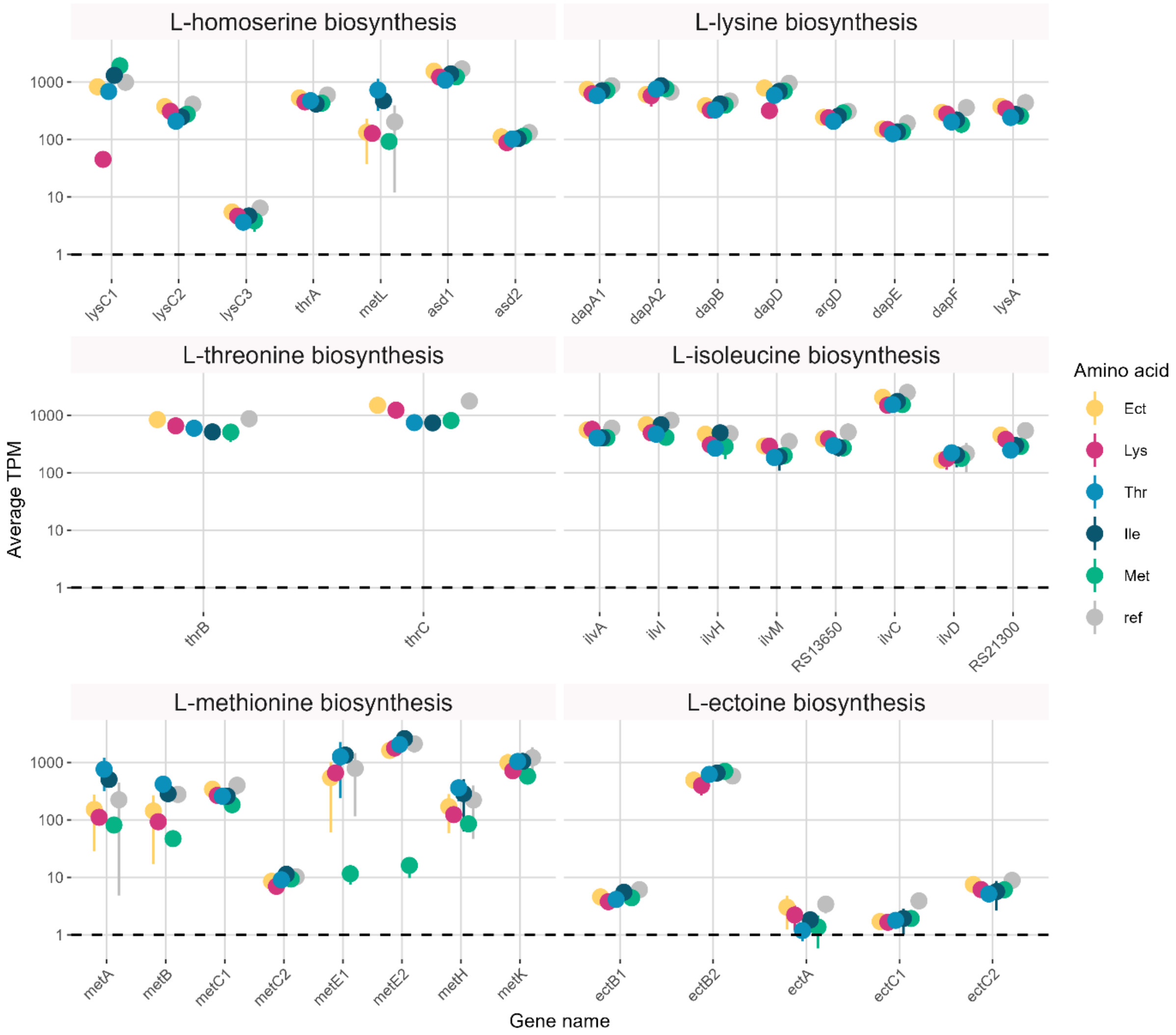

Figure S9: Absolute counts of genes implicated in L-lysine and related AFAAs biosynthesis pathway. Counts were normalized by gene length and library size. Normalized counts were then multiplied by 10^6 to obtain transcripts per million (TPM). Average TPM are given for the implicated genes for the reference and AFAAs supplementation experiments (plotted using R (ggplot2)).

Pathway	Enzymatic activity	Gene name	Gene id	0 mM NaCl	500 mM NaCl	800 mM NaCl
L-homoserine biosynthesis	Aspartate kinase (AK)	*lysC1*	*RS00270*	*		
		lysC2	*RS01175*	*	*	*
		lysC3	*RS05310*		*	*
	Aspartate kinase-homoserine dehydrogenase (AK-HD)	*thrA*	*RS11205*	*		
		metL	*RS14980*		*	*
	Aspartate-semialdehyde dehydrogenase	*asd1*	*RS03220*		*	
		asd2	*RS02955*	*	*	*
L-lysine biosynthesis	Dihydrodipicolinate synthase	*dapA1*	*RS02525*		*	*
		dapA2	*RS19840*			
	Dihydrodipicolinate reductase	*dapB*	*RS11310*			
	Tetrahydrodipicolinate succinylase	*dapD*	*RS02000*	*	*	*
	N-succinyl-diaminopimelate aminotransferase	*argD*	*RS14820*			
	N-succinyl-L-diaminopimelate desuccinylase	*dapE*	*RS02545*			
	Diaminopimelate epimerase	*dapF*	*RS14075*			
	Diaminopimelate decarboxylase	*lysA*	*RS14070*			
L-threonine biosynthesis	Homoserine kinase	*thrB*	*RS11200*	*		
	L-threonine synthase	*thrC*	*RS11195*	*		
L-isoleucine biosynthesis	L-threonine dehydratase	*ilvA*	*RS13635*	*		
	Acetohydroxyacid synthase	*ilvI*	*RS11720*		*	*
		ilvH	*RS11715*			
		ilvM	*RS13645*	*		
		RS13650	*RS13650*	*	*	*
	Acetohydroxyacid isomeroreductase	*ilvC*	*RS13345*			
	Dihydroxyacid dehydratase	*ilvD*	*RS13640*	*		
	Branched-chain amino acid aminotransferase	*RS21300*	*RS21300*		*	
L-methionine biosynthesis	Homoserine O-succinyltransferase	*metA*	*RS05150*	*	*	*
	O-succinylhomoserine-lyase	*metB*	*RS14975*		*	
	Cystathionine-β-lyase	*metC1*	*RS07710*		*	*
		metC2	*RS22415*	*	*	*
	Homocysteine transmethylase	*metE1*	*RS03895*	*		
		metE2	*RS19260*	*	*	*
		metH	*RS00265*	*	*	*
	L-methionine adenosyltransferase	*metK*	*RS00900*		*	*
L-ectoine biosynthesis	Diaminobutyrate pyruvate aminotransferase	*ectB1*	*RS05300*		*	*
		ectB2	*RS04065*	*		
	Diaminobutyrate acetyltransferase	*ectA*	*RS05295*		*	*
	Ectoine synthase	*ectC1*	*RS05305*		*	*
		ectC2	*RS16045*		*	

LFC: ≥ 2 / 0 / ≤ -2

Figure S10: Expression levels of the genes implicated in the L-lysine and related AFAAs biosynthesis pathways in *V. natriegens* under various NaCl concentrations. Colour indicates strength of the log2 fold change (LFC) of gene expression levels for each salt concentrations (0, 500 and 800 mM NaCl) compared to the reference condition (200 mM NaCl) (* – differentially expressed genes (DEGs)).

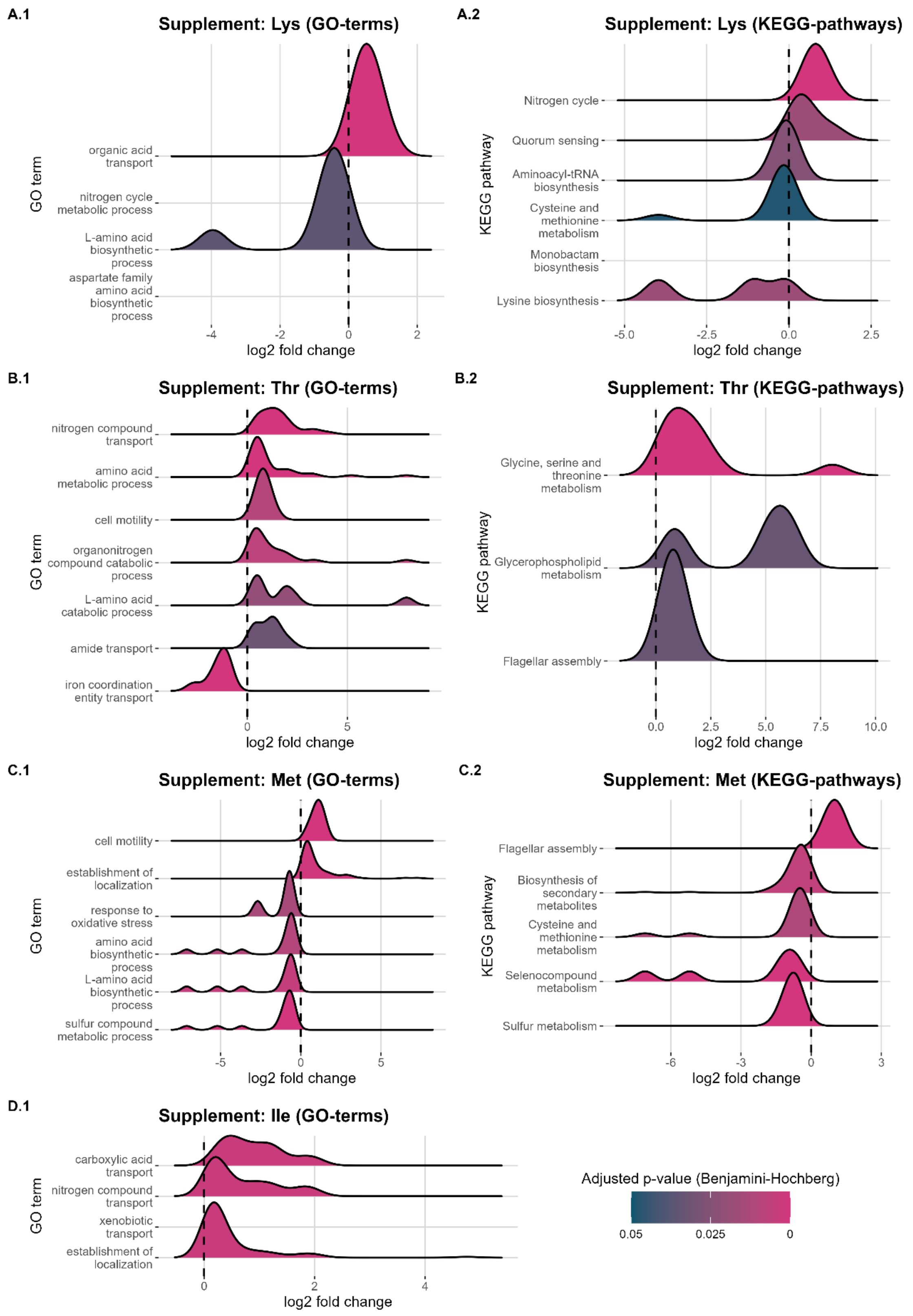

Figure S11: Gene set enrichment analysis of GO-terms (left) and KEGG pathways (right) gene sets for *V. natriegens* DSM759 *Δdns* cultures supplemented with L-methionine (A), L-lysine (B), L-threonine (C) and L-isoleucine (D). Each ridge represents the distribution of LFCs for core enriched GO-terms and KEGG pathways, respectively. Similar GO-terms were grouped. Ridge colour indicates the adjusted p-value for multiple testing (see legend). Ridges are not shown if the core enrichment genes consist of two genes or fewer.

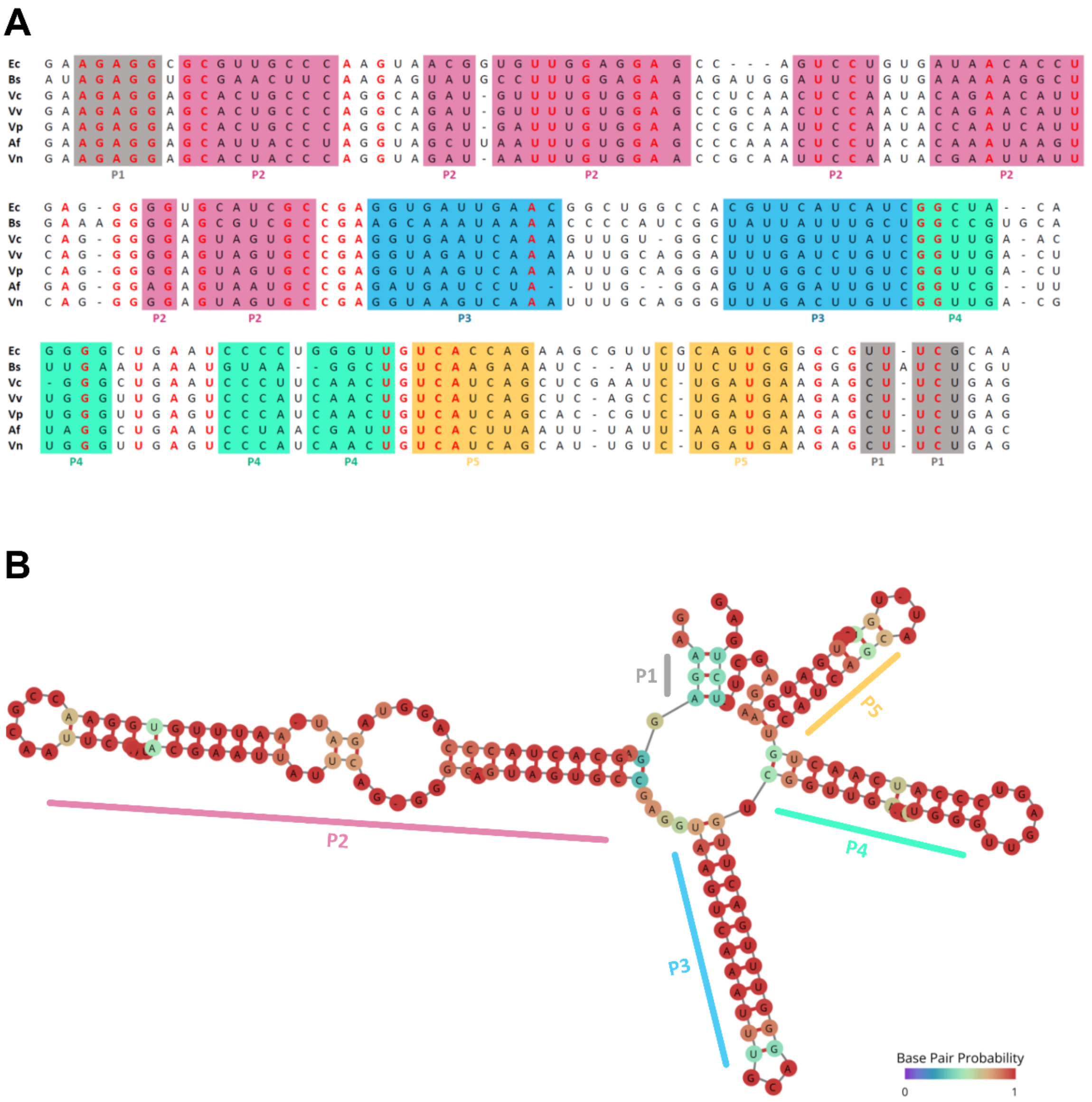

Figure S12: RNA sequence and secondary structure analysis of the putative L-lysine-sensitive *Vn.lysC1* riboswitch in *V. natriegens*. (A) Known riboswitch sequences upstream of *lysC* were taken from *E. coli* MG1655 (Ec), *B. subtilis* (Bs), *V. cholerae* (Vc), *Vibrio vulnificus serovar "E" str.* CECT 4999 (Vv), *Vibrio parahaemolyticus* FDAARGOS_115 (Vp) and *Aliivibrio_fischeri* ES114 (Af) and aligned with the *lysC1* upstream region of *V. natriegens* DSM759 (Vn) using ClustalX 2.1 [95]. Consensus bases were identified (highlighted red) and bases putatively participating in base-pairing elements of the conserved five-stem secondary RNA structure are individually coloured (P1, P2, P3, P4 and P5) [62]. **(B)** The predicted secondary RNA structure of the putative *Vn.lysC1* riboswitch is highly similar to that other *lysC* riboswitches reported in literature [61, 62]. The secondary structure was predicted using the ViennaRNA python package [96, 97].

A

																								T	I
V. natriegens	M	T	T	T	I	I	I	T	D	I	T	I	V	G	A	G	C	*						5	5
E. coli	M	K	R	I	S	T	T	I	T	T	T	I	T	I	T	T	G	N	G	A	G	*		8	4

B

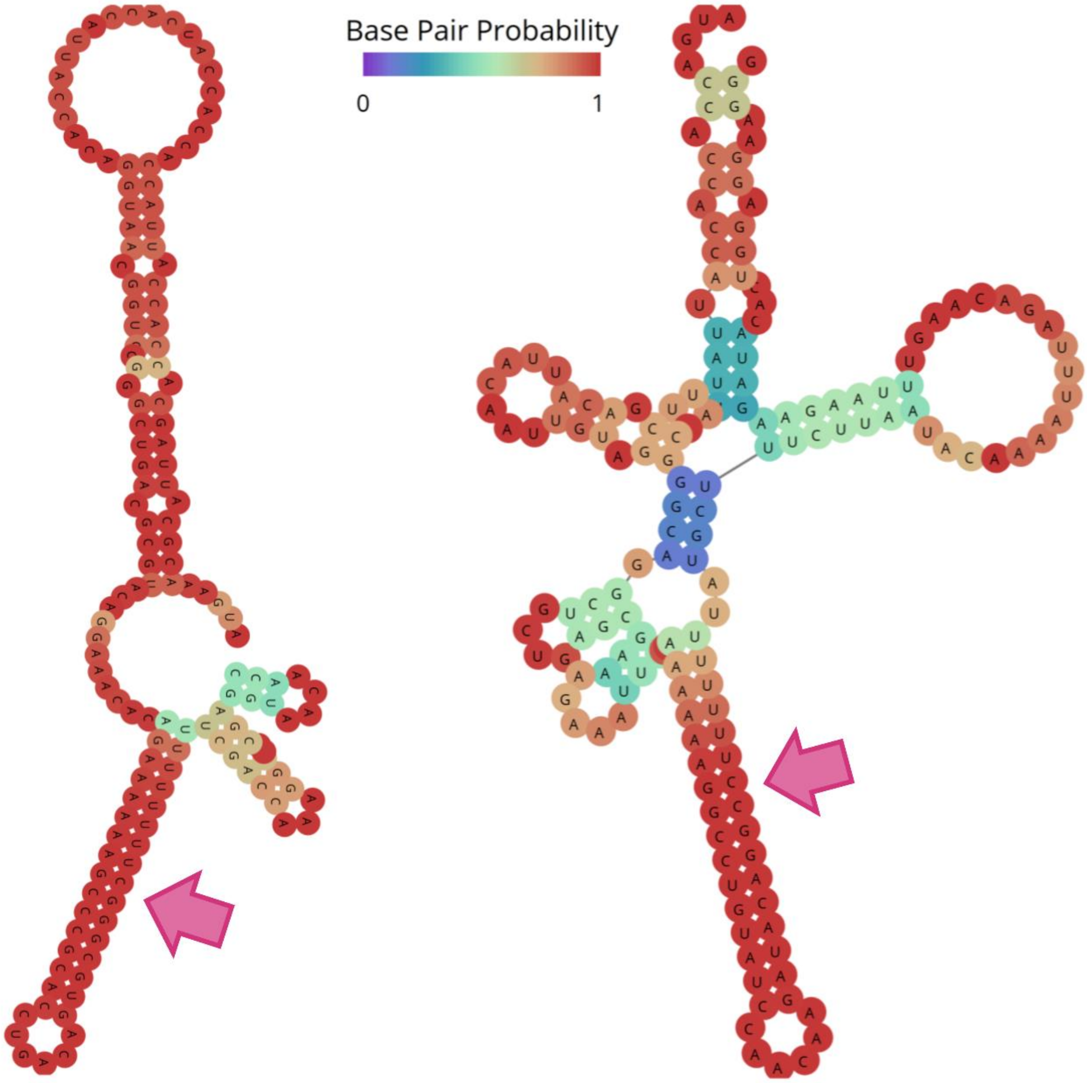

Figure S13: Analysis of the putative leader structure of the *thr* operon in *V. natriegens* and *E. coli* implicated in the transcriptional attenuation of the *thrABC* gene expression. (A) Comparison of the AA sequence of the putative *V. natriegens thr* leader peptide with that of *E. coli*. **(B)** Predicted secondary RNA structure of the 5' region of the *thr* operon in *E. coli* (left) and *V. natriegens* (right). The hairpin structure of the terminator, containing the typical poly-U motif, is highlighted (pink arrow).

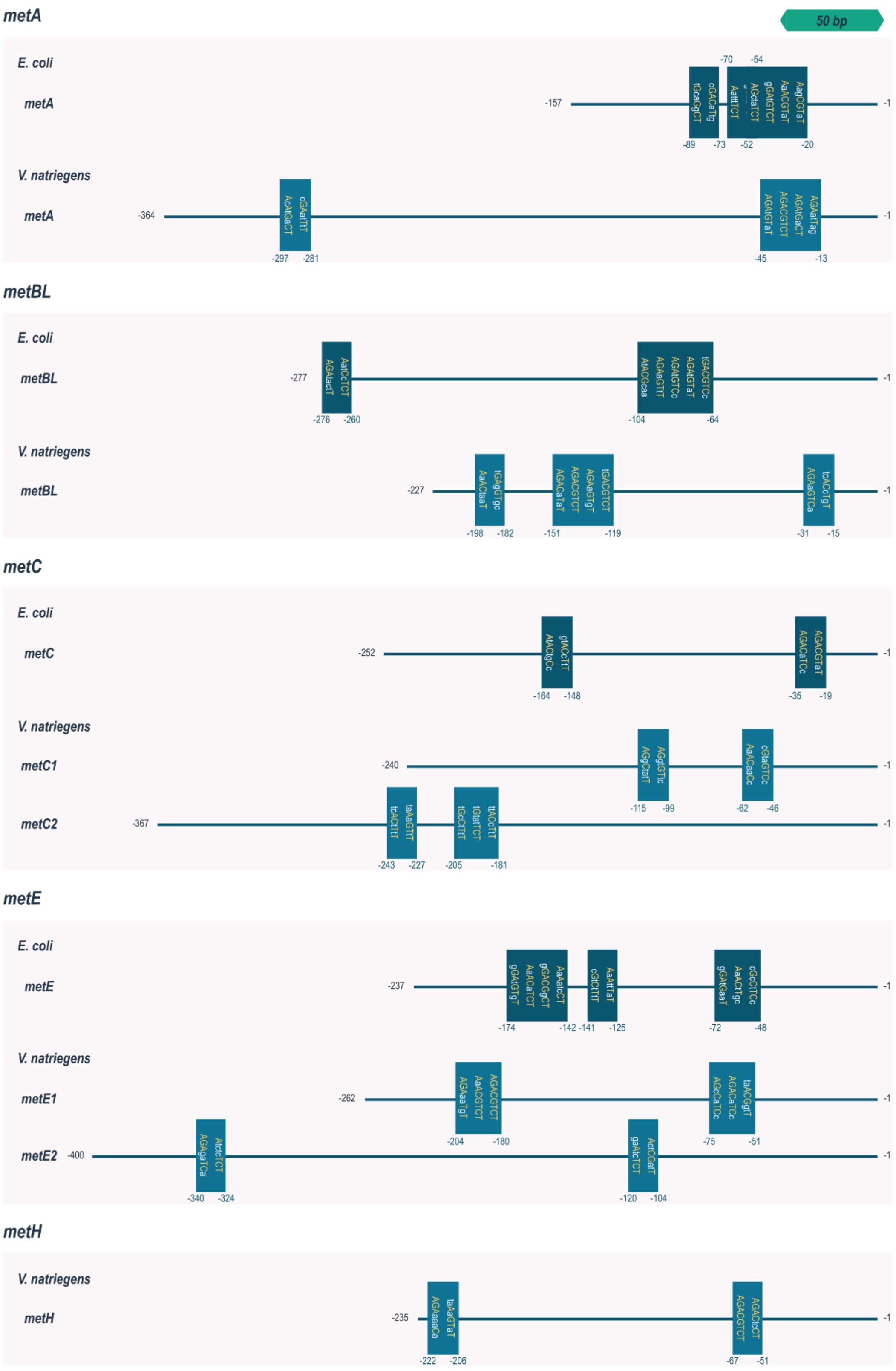

Figure S14: Putative *met* boxes within 5' sequences of *V. natriegens* and *E. coli met* genes. As the consensus sequence of *met* boxes, following eight-base DNA motif has been identified: "AGACGTCT". For *E. coli*, *met* genes generally have at least two to five contiguous *met* boxes, where an eight-base sequence has at least 50 % homology to the consensus motif [68]. Here, putative *met* boxes were identified within 5' sequences of *V. natriegens* DSM759 *met* genes *via* iterative search for at least two contiguous sequences matching the given parameters.
